# Human Cytomegalovirus IE1 Protein Elicits a Type II Interferon-Like Host Cell Response That Depends on Activated STAT1 but Not Interferon-γ

**DOI:** 10.1371/journal.ppat.1002016

**Published:** 2011-04-14

**Authors:** Theresa Knoblach, Benedikt Grandel, Jana Seiler, Michael Nevels, Christina Paulus

**Affiliations:** Institute for Medical Microbiology and Hygiene, University of Regensburg, Regensburg, Germany; Oregon Health and Science University, United States of America

## Abstract

Human cytomegalovirus (hCMV) is a highly prevalent pathogen that, upon primary infection, establishes life-long persistence in all infected individuals. Acute hCMV infections cause a variety of diseases in humans with developmental or acquired immune deficits. In addition, persistent hCMV infection may contribute to various chronic disease conditions even in immunologically normal people. The pathogenesis of hCMV disease has been frequently linked to inflammatory host immune responses triggered by virus-infected cells. Moreover, hCMV infection activates numerous host genes many of which encode pro-inflammatory proteins. However, little is known about the relative contributions of individual viral gene products to these changes in cellular transcription. We systematically analyzed the effects of the hCMV 72-kDa immediate-early 1 (IE1) protein, a major transcriptional activator and antagonist of type I interferon (IFN) signaling, on the human transcriptome. Following expression under conditions closely mimicking the situation during productive infection, IE1 elicits a global type II IFN-like host cell response. This response is dominated by the selective up-regulation of immune stimulatory genes normally controlled by IFN-γ and includes the synthesis and secretion of pro-inflammatory chemokines. IE1-mediated induction of IFN-stimulated genes strictly depends on tyrosine-phosphorylated signal transducer and activator of transcription 1 (STAT1) and correlates with the nuclear accumulation and sequence-specific binding of STAT1 to IFN-γ-responsive promoters. However, neither synthesis nor secretion of IFN-γ or other IFNs seems to be required for the IE1-dependent effects on cellular gene expression. Our results demonstrate that a single hCMV protein can trigger a pro-inflammatory host transcriptional response via an unexpected STAT1-dependent but IFN-independent mechanism and identify IE1 as a candidate determinant of hCMV pathogenicity.

## Author Summary

Human cytomegalovirus (hCMV) is a leading cause of birth defects and severe disease in people with compromised immunity. Disease caused by hCMV is frequently linked to inflammation, and the virus has been shown to induce numerous host genes many of which encode pro-inflammatory proteins. However, little is known about the contributions of individual viral proteins to these changes in cellular transcription. We systematically analyzed the effects of the hCMV immediate-early 1 (IE1) protein, a major viral transcriptional activator, on expression of >28,000 human genes. Following expression under conditions mimicking the situation during hCMV infection, IE1 elicited a transcriptional response dominated by the up-regulation of pro-inflammatory and immune stimulatory genes normally induced by the secreted signaling protein interferon-γ. However, IE1-mediated gene expression was independent of interferon induction, yet required the activated form of signal transducer and activator of transcription 1 (STAT1), a central mediator of interferon signaling. Indeed, STAT1 moved to the nucleus and became associated with IE1 target genes upon expression of the viral protein. Our results demonstrate that a single hCMV protein can trigger a pro-inflammatory host cell response via an unexpected mechanism and suggest that IE1 may contribute to hCMV disease in more direct ways than previously thought.

## Introduction

Human cytomegalovirus (hCMV), the prototypical β-herpesvirus, is an extremely widespread pathogen (reviewed in [1]). Primary hCMV infection is invariably followed by life-long viral persistence in all infected individuals. The groups most evidently affected by hCMV disease are humans with acquired or developmental immune deficits including allograft recipients receiving immunosuppressive drugs, human immunodeficiency virus-infected individuals, cancer patients undergoing intensive chemotherapy, and infants infected *in utero* (reviewed in [2]). In immunologically normal hosts, clinically relevant symptoms rarely accompany acute infections (reviewed in [3]), but viral persistence may contribute to chronic disease conditions including atherosclerosis, cardiovascular disease, inflammatory bowel disease, immune senescence, and certain malignancies (reviewed in [4], [5], [6], [7], [8]).

The pathogenesis of disease (e.g., pneumonitis, retinitis, hepatitis, enterocolitis, and encephalitis) associated with acute hCMV infection in immunocompromised people is most readily attributable to end organ damage either directly caused by cytopathic viral replication or by host immunological responses that target virus-infected cells. In contrast, chronic disease associated with persistent hCMV infection in immunocompetent individuals as well as in the allografts of transplant recipients is most likely related to prolonged inflammation (reviewed in [9]). In fact, hCMV has been frequently detected in the midst of intense inflammation, and a myriad of studies from transplant recipients and normal hosts have presented a strong case for this virus as an etiologic agent in chronic inflammatory processes, particularly those resulting in vascular disease (reviewed in [4]). At the molecular level, this is reflected by the fact that, in both human cells and animal models, cytomegalovirus infections activate numerous host genes many of which encode growth factors, cytokines, chemokines, and adhesion molecules with pro-inflammatory and immune stimulatory activities [10], [11], [12], [13], [14], [15], [16], [17], [18], [19], [20], [21], [22], [23]. A number of these virus-induced proteins are released from infected cells forming the viral “secretome” [4], [24], [25].

A large proportion of human genes that undergo activation during hCMV infection are normally controlled by interferons (IFNs) (reviewed in [26], [27]). The IFNs constitute a distinct group of cytokines synthesized and released by most vertebrate cells in response to the presence of many different pathogens including hCMV. They are divided among three classes: type I IFNs (primarily IFN-α and IFN-β), type II IFN (IFN-γ), and type III IFNs (IFN-λ or interleukin 28/29). The type I IFNs share many biological activities with type III IFNs, especially in host protection against viruses. IFN-γ, the sole type II IFN, is one of the most important mediators of inflammation and immunity exerting pleiotropic effects on activation, differentiation, expansion and/or survival of virtually any cell type of the immune system (reviewed in [28]). A significant body of research has identified the primary IFN pathway components and has characterized their roles in “canonical” signaling (reviewed in [29], [30]). In this pathway, IFNs bind to their cognate cell surface receptors to induce conformational changes that activate the receptor-associated enzymes of the Janus kinase (JAK) family. The post-translational modifications that follow this activation create docking sites for proteins of the signal transducer and activator of transcription (STAT) family with seven human members. In turn, the STAT proteins undergo JAK-mediated phosphorylation at a single tyrosine residue (Y701 in STAT1), which triggers their transition to an active dimer conformation. The STAT dimers accumulate in the nucleus where they may recruit additional proteins, and these complexes then bind sequence-specifically to short DNA motifs termed IFN-stimulated response element (ISRE) or gamma-activated sequence (GAS). ISREs are usually bound by a ternary complex composed of a STAT1-STAT2 heterodimer and IFN regulatory factor (IRF) 9, which forms upon induction by type I and type III IFNs and is referred to as IFN-stimulated gene factor 3 (ISGF3). In contrast, type II IFN typically signals via STAT1 homodimers that associate with GAS elements. Finally, promoter-associated STAT proteins stimulate transcription of numerous IFN-stimulated genes (ISGs) via their carboxy-terminal transcriptional activation domain. Within this domain, phosphorylation of a serine residue (S727 in STAT1) can augment STAT transcriptional activity. To some extent, the complex responses elicited by type I, type II, and type III IFNs are redundant as a consequence of partly overlapping ISGs.

Since many ISGs, especially those induced by type I IFNs, exhibit potent anti-viral activities most viruses have evolved escape mechanisms that mitigate IFN responses. In fact, both hCMV and murine cytomegalovirus (mCMV) are known to disrupt IFN pathways at multiple points (reviewed in [26], [27]). For example, JAK-STAT signaling is inhibited by the hCMV 72-kDa immediate-early 1 (IE1) gene product [31], [32], [33], a key regulatory nuclear protein required for viral early gene expression and replication in fibroblasts infected at low input multiplicities [34], [35], [36]. IE1 orthologs of mCMV and rat cytomegalovirus (rCMV) also contribute to replication and virulence in the respective animals [37], [38]. The hCMV IE1 protein counteracts virus- or type I IFN-induced ISG activation via complex formation with STAT1 and STAT2 resulting in reduced binding of ISGF3 to ISREs [31], [32], [33], [39]. STAT2 interaction contributes to hCMV type I IFN resistance and to IE1 function during productive infection [33], but the viral protein undergoes many additional host cell interactions (reviewed in [2], [40], [41]). For example, IE1 targets subnuclear structures known as promyelocytic leukemia (PML) bodies or nuclear domain 10 (ND10) ([42], [43], [44]; reviewed in [45], [46], [47], [48]). In addition, IE1 associates with chromatin [49] and interacts with a variety of transcription regulatory proteins [50], [51], [52], [53], [54], [55], [56], [57]. Consequently, IE1 stimulates expression from a broad range of viral and cellular promoters in transient transfection assays. However, IE1-mediated activation or repression of merely a few single endogenous human genes has been demonstrated so far [58], [59], [60], [61], [62], [63], [64].

Here we present the results of the first systematic human transcriptome analysis following expression of the hCMV IE1 protein. Surprisingly, the predominant response to IE1 was characterized by activation of pro-inflammatory and immune stimulatory genes normally controlled by IFN-γ. We further demonstrate that IE1 employs an unusual mechanism, which does not require induction of IFNs but nonetheless depends on activated (Y701-phosphorylated) STAT1, to up-regulate a subset of ISGs.

## Results

### Construction and characterization of human primary cells with inducible IE1 expression

The hCMV IE1 protein exhibits complex activities, and results obtained from experiments with IE1 mutant virus strains are inherently difficult to interpret. In fact, regarding the phenotype of IE1-deficient viruses at low input multiplicities, it seems almost impossible to discriminate between effects directly linked to any of the IE1 activities and indirect effects caused by delays in downstream viral gene expression and replication. On the other hand, following infection at high multiplicity, many consequences of absent IE1 expression are compensated for by excess viral structural components, such as tegument proteins and/or DNA, and therefore undetectable ([35], [36]; reviewed in [2], [40], [41]). Thus, it is apparent that cells with inducible expression of functional IE1 at physiological levels would be highly useful by allowing a definite assessment of the viral protein's activities outside the confounding context of infection. Furthermore, such cells would avoid potential difficulties typically associated with transient transfection, including variable frequency of positive cells and protein accumulation to non-physiologically high levels. Importantly, an inducible expression system would also preclude cells from adapting to long-term IE1 expression. In fact, the continued presence of IE1 is reportedly incompatible with genomic integrity and normal cell proliferation [65], [66], [67].

**Figure 1:**
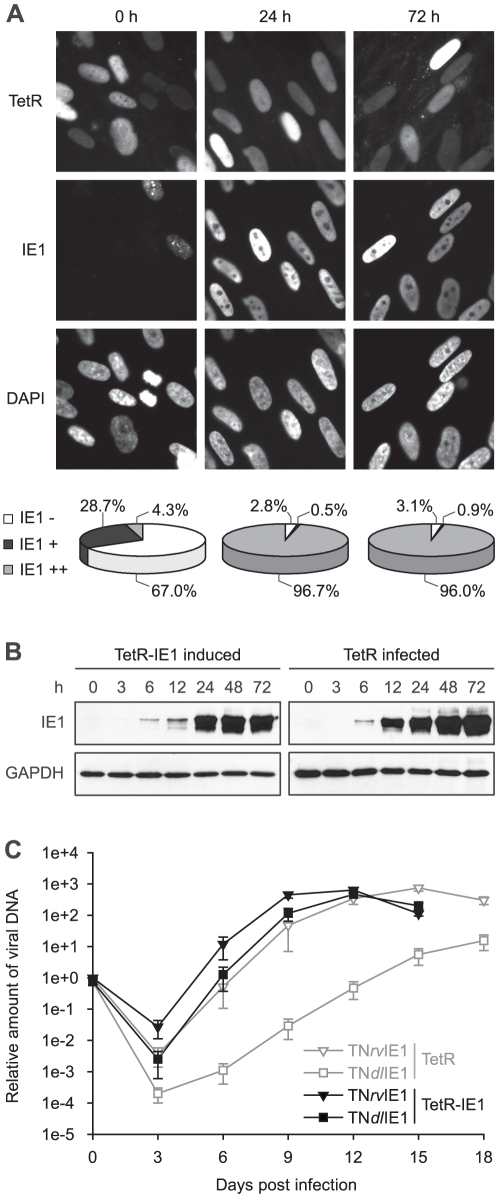
Characterization of TetR-IE1 cells. A) TetR-IE1 cells were treated with doxycycline for 24 and 72 h or were left untreated (0 h). Paraformaldehyde-fixed samples were examined by fluorescence microscopy for IE1 (antibody 1B12) and TetRnlsEGFP (TetR) expression (autofluorescence). Staining with 4′,6-diamidino-2-phenylindole (DAPI) was performed to visualize nuclei. Original magnification, ×504. For the pie charts, ∼500 randomly selected nuclei per sample were examined for IE1 expression. The scoring system is as follows: IE1 −, no IE1 staining above background; IE1 +, weak, mostly punctate IE1 staining; IE1 ++, strong, diffuse IE1 staining. B) Time course (0–72 h) immunoblot analysis of IE1 and GAPDH steady-state protein levels in doxycycline-induced TetR-IE1 cells and hCMV (TNwt)-infected TetR cells (MOI = 1 PFU/cell). To assure comparability between protein bands, gels loaded with extracts from equal cell numbers were run and blotted side by side under the same conditions, and pairs of membranes destined for IE1 or GAPDH detection were processed together and exposed on the same film. C) Multistep replication analysis of IE1-null mutant hCMV (TN*dl*IE1) and the corresponding revertant virus (TN*rv*IE1) in doxycycline-treated TetR and TetR-IE1 cells. Confluent cells were infected at an MOI of 0.01 PFU/cell, and viral replication was monitored at 3-day intervals by qPCR-based relative quantification of hCMV DNA from culture supernatants. Mean values and standard deviations of four independent infections with two different clones per each virus strain are shown.

We used a tetracycline-dependent induction (Tet-on) system built into lentivirus vectors to generate cells in which IE1 expression can be synchronously induced and compared to cells not expressing the viral protein. The first component of this system is a lentiviral vector (pLKOneo.CMV.EGFPnlsTetR; [68], [69], [70]) that includes a hybrid gene encoding the tetracycline repressor (TetR) linked to a nuclear localization signal (NLS) derived from the SV40 large T antigen and the enhanced green fluorescent protein (EGFP) to produce an EGFPnlsTetR fusion protein [68]. In addition, this vector encodes neomycin resistance. The second component is a lentivirus vector (pLKO.DCMV.TetO.cIE1) conferring puromycin resistance, in which a fragment of the hCMV promoter-enhancer drives expression of the IE1 (Towne strain) cDNA. In this vector, tandem tetracycline operator (TetO) sequences are present immediately downstream of the TATA box. For the lentivirus transductions, we chose MRC-5 primary human embryonic lung fibroblasts, because they support robust wild-type hCMV replication, whereas IE1-deficient virus strains exhibit a severe growth defect after low multiplicity infection of these cells ([31], [33] and Figure 1 C). Initially, low passage MRC-5 cells were transduced with lentivirus prepared from plasmid pLKOneo.CMV.EGFPnlsTetR, and a neomycin-resistant polyclonal cell population (named TetR) was isolated in which almost all cells expressed the EGFP fusion protein located in the nucleus (data not shown). Next, TetR cells were transduced with lentivirus prepared from pLKO.DCMV.TetO.cIE1 and a mixed cell population (named TetR-IE1) exhibiting both neomycin and puromycin resistance was selected. Finally, fluorescence-activated cell sorting was performed to collect cells with high levels of EGFPnlsTetR and, consequently, low levels of IE1 in the absence of inductor.

To characterize the newly generated cells, TetR-IE1 cells were treated with doxycycline for 24 or 72 h and examined for IE1 expression by indirect immunofluorescence microscopy (Figure 1 A). Before induction, the majority (67.0%) of cells was IE1 negative, and most other cells expressed barely detectably levels of the viral protein. Interestingly, in the latter proportion of cells IE1 was present in a predominantly punctate nuclear pattern. This likely reflects stable co-localization between IE1 and ND10 due to viral protein levels insufficient to disrupt the nuclear structures. At 24 h following induction only 2.8% of cells were negative for IE1 expression and >97% stained positive for the viral protein. In almost all positive cells IE1 exhibited a largely diffuse nuclear staining indicating complete disruption of ND10. Very similar results were obtained for IE1 expression and localization 72 h post induction. Importantly, the observed temporal and spatial pattern of IE1 subnuclear localization in TetR-IE1 cells closely resembles that observed during productive hCMV infection in fibroblasts where initial colocalization between IE1 and ND10 is succeeded by ND10 disruption and diffuse nuclear distribution of the viral protein [43], [44], [71].

To compare the relative levels of IE1 expressed during hCMV infection and after induction of TetR-IE1 cells, TetR cells were infected with the hCMV Towne strain, and samples collected before or 3 h, 6 h, 12 h, 24 h, 48 h and 72 h after infection were analyzed for IE1 steady-state protein levels in comparison with samples of TetR-IE1 cells that had been treated with doxycycline (Figure 1 B). The timing of IE1 induction in TetR-IE1 cells was remarkably similar to the kinetics of IE1 accumulation in hCMV-infected cells. In addition, the IE1 levels detected at 24 to 72 h post induction were comparable to the protein amounts that had accumulated by 24 h post hCMV infection.

To confirm that TetR-IE1 cells express fully active IE1, replication of wild-type and IE1-deficient hCMV strains was compared by multi-step analyses conducted in doxycycline-treated TetR and TetR-IE1 cells (Figure 1 C). To this end, we employed a bacterial artificial chromosome (BAC)-based recombination approach to generate a “markerless” mutant virus strain (TN*dl*IE1) lacking the entire IE1-specific coding sequence. For details on the construction of TN*dl*IE1 and a revertant virus (TN*rv*IE1) see Materials and Methods. As expected, the replication of two independent TN*dl*IE1 clones was strongly attenuated in TetR cells, with a ∼2 to >3 log difference in titers between mutant and revertant virus strains. It is important to note that our previous work has shown that TN*rv*IE1 and the parental wild-type strain (TNwt) exhibit identical replication kinetics [33]. However, induced TetR-IE1 cells were able to support wild-type-like replication of the TN*dl*IE1 viruses demonstrating that the viral protein provided in *trans* can fully compensate for the lack of IE1 expression from the hCMV genome during productive infection. Interestingly, even the titers of TN*rv*IE1 were reproducibly up to ∼20-fold higher in TetR-IE1 as compared to IE1-negative cells between 3 and 12 days post infection.

Taken together, these results show that in TetR-IE1 cells expression of IE1 can be synchronously induced from the autologous hCMV major IE (MIE) promoter resulting in fully functional protein at levels present during the early stages of hCMV infection. Thus, TetR/TetR-IE1 cells present an ideal model to study the activities of the IE1 protein outside the complexity of infection, yet under physiological conditions.

### IE1 triggers a pro-inflammatory and immune stimulatory human transcriptome response

The capacity of hCMV IE1 to activate transcription from both viral and cellular promoters has long been appreciated ([72]; reviewed in [2], [40], [41]). However, most reports on IE1-regulated host gene transcription have relied on transient transfections and promoter-reporter assays. To our knowledge, regulation of endogenous cellular transcription by IE1 has so far only been studied sporadically and at the level of single genes.

To comprehensively assess the impact of IE1 on the human transcriptome, we performed a systematic gene expression analysis using our TetR/TetR-IE1 cell model and Affymetrix GeneChip Human Gene 1.0 ST Arrays covering 28,869 genes (>99% of sequences currently present in the RefSeq database, National Center for Biotechnology Information). We compared the gene expression profiles at 24 h and 72 h post induction in induced versus non-induced TetR-IE1 cells and in induced TetR-IE1 versus induced TetR cells. Expression from the vast majority (99.9%) of genes represented on the arrays was not significantly affected by IE1. However, mRNA levels of 38 human genes differed by a factor of two or more (*p*>0.01) in both the induced TetR-IE1/non-induced TetR-IE1 and the induced TetR-IE1/induced TetR comparisons. For 32 (84%) of the 38 genes, changes in mRNA levels were only observed after 72 h (but not 24 h) of IE1 expression, and only six (16%) were differentially expressed at both 24 h and 72 h. Moreover, 13 (34%) of these genes were down-regulated by a factor between 2.0 and 5.5 (data not shown) and 25 (66%) were up-regulated by a factor between 2.0 and 41.9 (Table 1). For the present work, we concentrated on the set of genes that was found to be up-regulated by expression of IE1.

**Table 1:** Human genes with increased mRNA levels after IE1 induction.

Gene		Maximum fold increase			
		24 h post induction		72 h post induction	
ID	Symbol	IE1+/TetR+	IE1+/IE1−	IE1+/TetR+	IE1+/IE1−
8995	TNFSF18	9.0	2.6	12.6	4.8
7292	TNFSF4	6.2	2.1	6.5	2.5
3627	CXCL10	3.5	2.4	41.9	24.6
27063	ANKRD1	3.3	2.3	10.1	8.3
1906	EDN1	2.4	1.9	3.3	3.6
3620	IDO1	1.6	1.1	28.7	20.2
115361	GBP4	1.7	1.2	17.3	13.5
6373	CXCL11	1.4	1.1	13.3	10.5
115362	GBP5	1.1	1.0	7.5	7.0
10964	IFI44L	1.3	1.1	4.6	4.4
4283	CXCL9	1.3	1.0	4.5	4.2
29126	CD274	1.2	1.5	3.9	4.5
3122	HLA-DRA	1.2	1.1	3.4	3.5
2633	GBP1	1.5	1.2	3.1	2.7
3433	IFIT2	1.4	1.0	2.9	2.0
6356	CCL11	1.7	1.2	2.8	2.2
3280	HES1	1.7	1.3	2.6	2.2
56256	SERTAD4	1.4	1.1	2.6	2.0
2634	GBP2	−1.1	−1.1	2.5	3.9
1520	CTSS	1.0	1.0	2.5	2.2
3047	HBG1	1.2	1.0	2.4	2.1
3659	IRF1	1.2	1.3	2.3	2.5
6890	TAP1	1.2	1.1	2.3	2.1
83643	CCDC3	1.1	1.1	2.3	2.1
3437	IFIT3	−1.1	1.9	2.1	2.1

IE1+, doxycycline-treated TetR-IE1 cells; TetR+, doxycycline-treated TetR cells; IE1−, non-induced TetR-IE1 cells.

**Figure 2:**
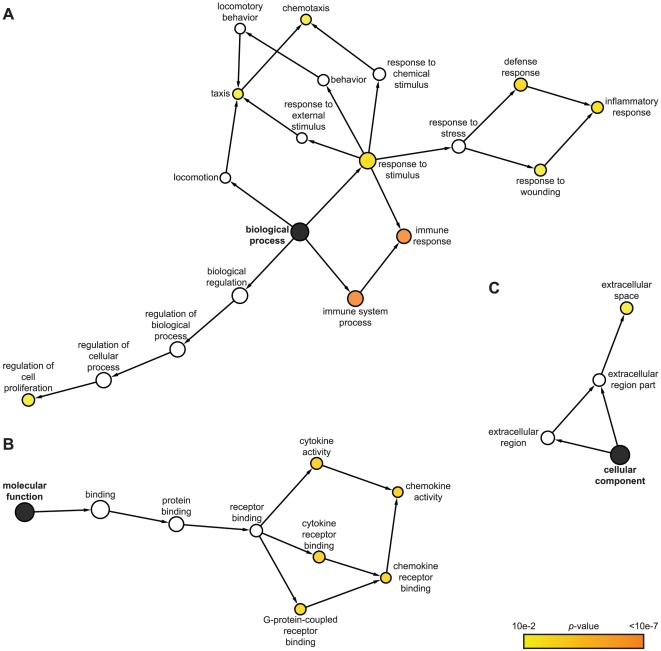
Predominant functional themes among IE1-activated genes. Cytoscape (http://www.cytoscape.org [219], [220]) and the Biological Networks Gene Ontology (BiNGO) plugin (http://www.psb.ugent.be/cbd/papers/BiNGO [221]) were used to map and visualize overrepresented terms in the IE1-activated human transcriptome on the GO hierarchy. Spatial arrangement of nodes reflects grouping of categories by semantic similarity. The node area is proportional to the number of genes in the reference set (“GO Full”, *Homo sapiens*) annotated to the corresponding GO term. The yellow to orange node color indicates how significantly individual terms are overrepresented (*p*≤0.01; hypergeometric test including Benjamini and Hochberg False Discovery Rate correction [222]). White nodes are included to show the colored nodes in the context of the GO hierarchy and are not significantly overrepresented. Black nodes represent the three GO domains: A) biological process, B) molecular function, and C) cellular component.

We utilized the Gene Ontology (GO) classification system (http://www.geneontology.org) to identify attributes which predominate among IE1-activated gene products regarding the three GO domains “biological process”, “molecular function”, and “cellular component”. Furthermore, we employed a set of analysis tools to construct maps that visualize overrepresented attributes on the GO hierarchy (Figure 2). According to GO, the most significantly enriched “biological process” terms with respect to the 25 IE1-activated genes are: “immune system process”, “immune response”, “inflammatory response”, “response to wounding”, “response to stimulus”, “defense response”, “chemotaxis”, “taxis”, and “regulation of cell proliferation” (Figure 2 A). In fact, virtually all IE1-induced genes with assigned functions have been implicated in adaptive or innate immune processes including inflammation. Moreover, 7 (28%) of the 25 genes encode known cytokines or other soluble mediators, namely the chemokine (C-X-C motif) ligands CXCL9, CXCL10 and CXCL11, the chemokine (C-C motif) ligand CCL11, endothelin 1 (encoded by EDN1), and the tumor necrosis factor (TNF) superfamily members 4 (TNFSF4, also known as OX40 ligand) and 18 (TNFSF18, also known as GITR ligand). This observation is also illustrated by the fact that, according to GO, the most significantly enriched “molecular function” terms in the IE1-activated transcriptome are: “cytokine receptor binding”, “cytokine activity”, “chemokine activity”, “chemokine receptor binding”, and “G-protein-coupled receptor binding” (Figure 2 B). Furthermore, the top “cellular component” category is “extracellular space” (Figure 2 C). For a more thorough assessment of overrepresented GO terms among IE1-induced genes, see Supporting Tables S1, S2 and S3.

Surprisingly, the genes induced by IE1 are generally associated with stimulatory rather than inhibitory effects on immune function including inflammation (Figure 2 A and Supporting Table S1). For example, some of the gene products are involved in the proteolysis (cathepsin S encoded by CTSS), intracellular transport (TAP1 transporter) or cell surface presentation (HLA-DRA) of antigens (reviewed in [73]). The chemokines CXCL9, CXCL10, and CXCL11 mediate leukocyte migration (see Discussion; reviewed in [73], [74], [75]). CD274 (also known as PDL1), TNFSF4, and TNFSF18 are co-stimulatory molecules which promote leukocyte (including T and B lymphocyte) activation, proliferation and/or survival (reviewed in [73], [76], [77], [78], [79]). Indoleamine 2,3-dioxygenase 1 (IDO1) and IRF1 have also been linked to T lymphocyte regulation, but they have additional functions in innate immune control of viral infection (reviewed in [73], [80], [81], [82], [83], [84], [85]. Likewise, GBP1 and murine GBP2 exhibit antiviral activity [86], [87], [88], [89].

**Figure 3:**
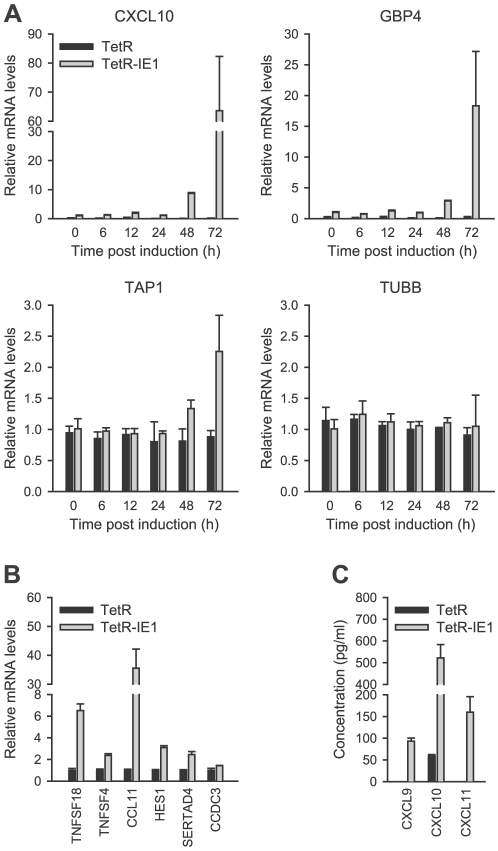
Confirmation of IE1-induced gene expression. A) TetR and TetR-IE1 cells were treated with doxycycline for 0 to 72 h as indicated. Relative mRNA expression levels were determined by qRT-PCR with primers specific for the CXCL10, GBP4, TAP1, and TUBB genes. Means and standard deviations of two replicates are shown in comparison to uninduced TetR-IE1 cells (set to 1). B) TetR and TetR-IE1 cells were treated with doxycycline for 72 h. Relative mRNA expression levels were determined by qRT-PCR with primers specific for the indicated genes. Means and standard deviations of two biological and two technical replicates are shown in comparison to TetR cells (set to 1). C) Quantification of the CXCR3 ligands CXCL9, CXCL10, and CXCL11 in the supernatant of IE1 expressing cells. Growth-arrested TetR and TetR-IE1 cells were treated with doxycycline for 72 h. The culture medium was replaced by 0.5 volumes of doxycycline containing DMEM with 0.1% BSA, and chemokine protein accumulation was determined 24 h later by quantitative sandwich enzyme immunoassay. Means and standard deviations of two biological and two technical replicates are shown.

**Figure 4 ppat-1002016-g004:**
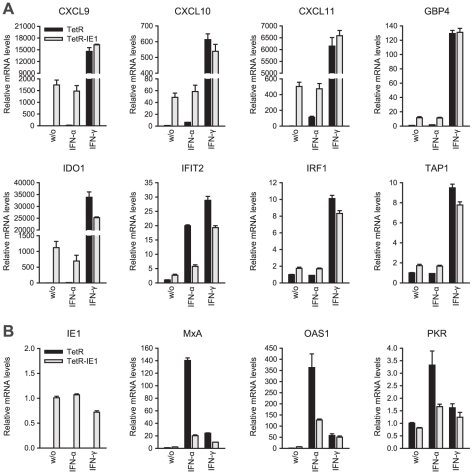
IE1 induces an IFN-γ-like transcriptional response. TetR and TetR-IE1 cells were treated with doxycycline for 72 h and solvent (w/o), IFN-α, or IFN-γ for 24 h. Relative mRNA expression levels were determined by qRT-PCR with primers specific for a set of IE1-responsive genes (A), typical type I IFN response genes and IE1 itself (B). Results were normalized to TUBB, and mean values with standard deviations from two biological and two technical replicates are shown. ISG expression is shown in comparison to untreated TetR cells (set to 1). IE1 expression is presented relative to untreated TetR-IE1 cells (set to 1).

Out of the 25 IE1-activated genes, 14 were selected for validation by qRT-PCR. The selected genes were representative of the entire range of expression kinetics and induction magnitudes measured by microarray analysis. The PCR approach confirmed expression of all tested genes typically reporting similar or larger fold increases compared to the array data (Figure 3 A–B and Figure 4 A). For example, in induced (72 h) versus non-induced TetR-IE1 cells the CXCL10 mRNA was found to be increased 24.6-fold by array analysis (Table 1) and 68.0-fold by PCR (Figure 3 A). Under the same conditions, the GBP4 transcript was induced 13.5-fold by array analysis (Table 1) as compared to 19.1-fold by PCR (Figure 3 A). The corresponding data for TAP1 were 2.1-fold (array analysis; Table 1) and 2.3-fold (PCR; Figure 3 A). Largely concordant results regarding induction magnitudes between array and PCR analyses were also obtained for CCDC3, CCL11, HES1, SERTAD4, TNFSF4, and TNFSF18 (Figure 3 B) as well as for CXCL9, CXCL11, IDO1, IFIT2, and IRF1 (Figure 4 A). In addition to the extent of gene activation, the precise timing of induction was exemplary investigated for CXCL10, GBP4 and TAP1 (Figure 3 A). A substantial increase in mRNA production from all three genes was evident at 72 h (and to a lesser extent at 48 h) but only minor effects were detected between 6 h and 24 h post IE1 induction consistent with the array data (Table 1). Tubulin-β (TUBB) gene expression, which is not affected by IE1, served as a negative control for the PCR experiments. Finally, the chemokines CXCL9 and CXCL11 were exclusively detected in supernatants from TetR-IE1 but not TetR cells (Figure 3 C). Moreover, the levels of CXCL10 protein were drastically increased in TetR-IE1 compared to TetR cells. This demonstrates that for these genes elevated mRNA levels also translate into enhanced protein synthesis and secretion.

The fact that increased expression of all tested IE1-activated genes was detectable with two or three alternative approaches strongly suggests that essentially all genes identified within the given experimental framework and data analysis settings are truly differentially expressed upon induction of IE1. Moreover, the activation of at least a subset of IE1-responsive genes appears to be temporally coupled.

### Most IE1-activated genes are ISGs normally controlled by IFN-γ

A plethora of past studies has established that immune regulatory genes are preferential targets of IFN-based regulation [28], [29], [30]. Intriguingly, at least 21 (84%) of the 25 IE1-activated human genes identified by microarray analysis turned out to be *bona fide* ISGs (Table 2) according to informations retrieved from the Interferome database (http://www.interferome.org [90]) and other sources including our own qRT-PCR analyses (Figure 4 A and Supporting Table S4). Several of these ISGs cluster in certain chromosomal locations (e.g., 1p22, 4q21, and 10q23-q25; Table 2) apparently reflective of their co-regulation.

**Table 2:** Genomic location and IFN responsiveness of IE1-induced human genes.

Gene		IFN-responsive		
Symbol	Locus	Yes/No	Type	Reference
IFI44L	1p31.1	Yes	I, II, III	Interferome^1^
GBP1	1p22.2	Yes	I, II, III	Interferome
GBP2	1p22.2	Yes	I, II	Interferome
GBP4	1p22.2	Yes	I, II	Interferome
			II	Figure 4 A
GBP5	1p22.2	Yes	I, III	Interferome
			II	[223]
CTSS	1q21	Yes	I	Interferome
			II	[148]
TNFSF18	1q23	Yes	II	Interferome
			−2	Supporting Table S4
TNFSF4	1q25	No	−	Interferome
			−2	Supporting Table S4
SERTAD4	1q32.1-q41	No	−	Interferome
			−	Supporting Table S4
HES1	3q28-29	Yes	−	Interferome
			II	Supporting Table S4
CXCL9	4q21	Yes	I, II	Interferome
			I, II	Figure 4 A
CXCL10	4q21	Yes	II	Interferome
			I, II	Figure 4 A
CXCL11	4q21.2	Yes	I, II	Interferome
			I, II	Figure 4 A
IRF1	5q31.1	Yes	I, II, III	Interferome
			II	Figure 4 A
EDN1	6p24.1	Yes	II	Interferome
HLA-DRA	6p21.3	Yes	I, II	Interferome
TAP1	6p21.3	Yes	I, II, III	Interferome
			II	Figure 4 A
IDO1	8p12-11	Yes	I, II	Interferome
			I, II	Figure 4 A
CD274	9p24	Yes	II	Interferome
CCDC3	10p13	No	−	Interferome
			−	Supporting Table S4
IFIT2	10q23-q25	Yes	I, II, III	Interferome
			I, II	Figure 4 A
IFIT3	10q24	Yes	I, II, III	Interferome
ANKRD1	10q23.31	Yes	I, II	Interferome
HBG1	11p15.5	No	−	Interferome
			−	Supporting Table S4
CCL11	17q21.1-21.2	Yes	−	Interferome
			II	Supporting Table S4

1 [90].

2 Marginally (≥1,5-fold) induced by IFN-α and/or IFN-γ (Supporting Table S4).

An initial assessment mainly based on the Interferome data revealed that IE1-activated ISGs are normally induced by either only IFN-γ or by both type II and type I IFNs (Table 2). To confirm this assignment and to further discriminate between type I and type II ISGs, we treated TetR and TetR-IE1 cells with exogenous IFN-α or IFN-γ and analyzed the effects on mRNA accumulation from a select subset of IE1-responsive ISGs. The transcript levels of all tested ISGs, namely CXCL9–11, GBP4, IDO1, IFIT2, IRF1, and TAP1 (Figure 4 A) as well as CCL11 (Supporting Table S4) were not only increased by IE1 expression (TetR-IE1 relative to TetR cells) but also by IFN-γ treatment of TetR cells, although to varying degrees (∼2 to >30,000-fold; Figure 4 A). Notably, there was a significant positive correlation (Pearson's correlation coefficient = 0.81) between the magnitudes of IE1- and IFN-γ-mediated ISG induction. In contrast, the same genes were substantially less susceptible (CXCL9–11, GBP4, IDO1, and IFIT2) or entirely unresponsive (CCL11, IRF1, and TAP1) to IFN-α (Figure 4 A), and there was no correlation (Pearson's correlation coefficient = −0.04) between IE1 and IFN-α responsiveness. For comparison, three typical type I ISGs, the genes encoding eukaryotic translation initiation factor 2α kinase 2 (EIF2AK2, also known as PKR), myxovirus (influenza virus) resistance 1 (Mx1, also known as MxA), and 2′,5′-oligoadenylate synthetase (OAS1), were strongly induced by IFN-α but barely by IFN-γ or IE1 (Figure 4 B). Although no obvious synergistic or additive effects between IE1 expression and IFN-γ treatment were observed in these assays (Figure 4 A–B), IFN-α induction of type I ISGs was severely compromised in TetR-IE1 as compared to TetR cells (Figure 4 B). The latter observation is consistent with our previous work which has demonstrated that IE1 blocks STAT2-dependent signaling resulting in inhibition of type I ISG activation [31], [33].

Hence, it appears that expression of IE1 selectively activates a subset of ISGs and ISG gene clusters which are primarily responsive to IFN-γ indicating that the viral protein elicits a type II IFN-like transcriptional response.

### IE1-mediated ISG activation is independent of IFNs

ISG activation typically requires synthesis, secretion and receptor binding of IFNs (reviewed in [26], [27], [29], [30]). IFN-α is encoded by a multi-gene family and is mainly expressed in leukocytes although some members are stimulated by IFN-β in fibroblasts [91]. However, neither of 12 IFN-α (IFNA) and three alternative type I IFN coding genes (IFNE, IFNK, and IFNW1 encoding IFN-ε, IFN-κ, and IFN-ω, respectively) was noticeably induced by IE1 as judged by our microarray results (Supporting Table S5). In contrast to IFN-α, IFN-β is encoded by a single gene (IFNB) and is produced by most cell types, especially by fibroblasts (IFN-β is also known as “fibroblast IFN”). However, previous work has shown that IE1 expression does not induce transcription from the IFN-β gene in fibroblasts [31], [32], [92]. Consistently, our microarray data did not reveal appreciable differences in IFNB1 mRNA levels between TetR and TetR-IE1 cells (Supporting Table S5). The single human IFN-γ gene (IFNG) is expressed upon stimulation of many immune cell types but not usually in fibroblasts, and our microarray results indicate that IE1 does not activate expression from this gene. Likewise, none of the known type III IFN genes (IL28A, IL28B, and IL29 encoding IFN-λ2/IL-28A, IFN-λ3/IL-28B, and IFN-λ1/IL-29, respectively) was significantly responsive to IE1 expression in this system (Supporting Table S5). For the IFN-β and IFN-γ transcripts, these results were confirmed by highly sensitive qRT-PCR from doxycycline-treated TetR-IE1 and TetR cells. Levels of the two IFN mRNAs did not significantly differ between TetR-IE1 and TetR cells at any of ten post induction time points (0 h–96 h) under investigation (Supporting Figure S1 and Supporting Table S6). Thus, IE1 does not seem to induce expression from the IFN-γ or any other human IFN gene.

**Figure 5 ppat-1002016-g005:**
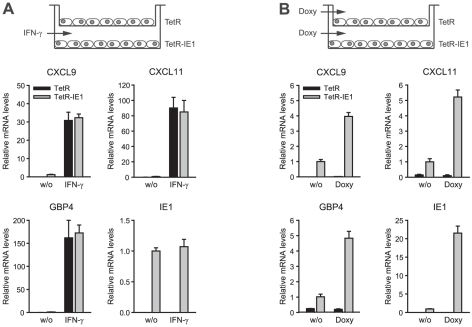
ISG induction is limited to IE1 expressing cells. TetR and TetR-IE1 cells were placed in the upper and lower chambers, respectively, of transwell dishes. Cells were growth-arrested and then treated in one of two ways. A) TetR-IE1 cells in the bottom chambers were treated with IFN-γ for 24 h or were left untreated (w/o). B) TetR cells in the upper and TetR-IE1 cells in the lower chambers were treated with doxycycline (Doxy) for 72 h or were left untreated (w/o). RNA was prepared from each compartment and analyzed by qRT-PCR with primers for the CXCL9, CXCL11, GBP4, and IE1 genes. Results were normalized to TUBB and mean values with standard deviations from two biological and two technical replicates are shown in comparison to untreated TetR-IE1 cells (set to 1).

To further rule out the possibility that ISG activation is a result of low level IFN production or secretion of any other soluble mediator from IE1 expressing cells, culture supernatants from TetR-IE1 cells induced with doxycycline for 24 h or 72 h were transferred to MRC-5 cells. As expected, MRC-5 cells did not undergo ISG induction 3 h to 72 h following media transfer (data not shown). Furthermore, we set up a transwell system with TetR cells in the top and TetR-IE1 cells in the bottom chamber (Figure 5). Following addition of IFN-γ to the lower chamber, we observed substantially increased mRNA levels of three IE1-responsive indicator ISGs (CXCL9, CXCL11, and GBP4) in both TetR and TetR-IE1 cells (Figure 5 A). In contrast, addition of doxycycline caused up-regulation of ISG mRNA levels in TetR-IE1 but not TetR cells (Figure 5 B). These results indicate that ISG induction is restricted to IE1 expressing cells and that a diffusible factor is not sufficient to mediate gene activation by the viral protein.

**Figure 6 ppat-1002016-g006:**
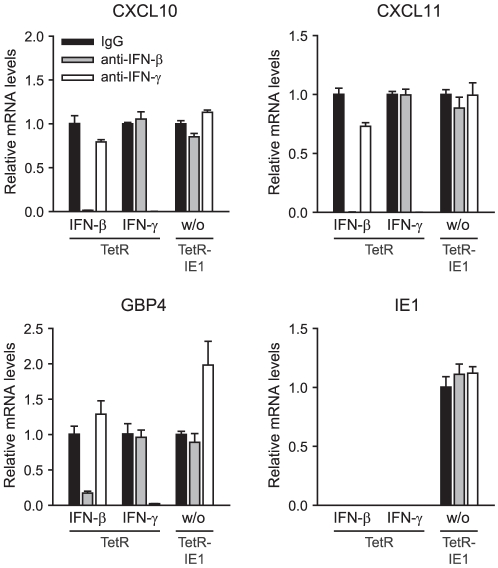
Presence of IFN-β- and IFN-γ-neutralizing antibodies does not impair ISG induction by IE1. TetR and TetR-IE1 cells were treated with doxycycline for 72 h and with solvent (w/o), IFN-β or IFN-γ for 24 h. Doxycycline and IFN treatment was performed in the continuous presence of normal goat immunoglobulin G (IgG), goat anti-IFN-β or goat anti-IFN-γ antibodies. Relative mRNA expression levels were determined by qRT-PCR with primers specific for the CXCL10, CXCL11, GBP4, and IE1 genes. Results were normalized to TUBB and mean values with standard deviations from two biological and two technical replicates are shown. Expression is shown in comparison to normal IgG-treated cells (set to 1).

Finally, we performed experiments adding neutralizing antibodies directed against IFN-β and IFN-γ to the cell culture media (Figure 6). ISG-specific qRT-PCRs from TetR cells treated with a combination of antibodies and high doses of the respective exogenous IFN confirmed that cytokine neutralization was both highly effective and specific. At the same time, neither the IFN-β- nor the IFN-γ-specific neutralizing antibodies had any significant negative effect on IE1-mediated ISG induction. These results strongly support the view that ISG activation by IE1 is independent of IFN-β, IFN-γ, and likely other IFNs.

### IE1-mediated ISG activation depends on STAT1 but not STAT2

Homodimeric STAT1 complexes are the central intracellular mediators of canonical IFN-γ signaling (reviewed in [26], [27], [28], [29], [30]). Interestingly, previous work has shown that the IE1 protein interacts with both STAT1 and STAT2, although STAT2 binding appeared to be more efficient [31], [32], [33], [39]. STAT2 has also been implicated in certain IFN-γ responses ([93], [94]; reviewed in [95]), although some (hCMV-mediated) activation of ISG transcription appears to occur entirely independent of STAT proteins ([96]; reviewed in [26], [27]).

**Figure 7:**
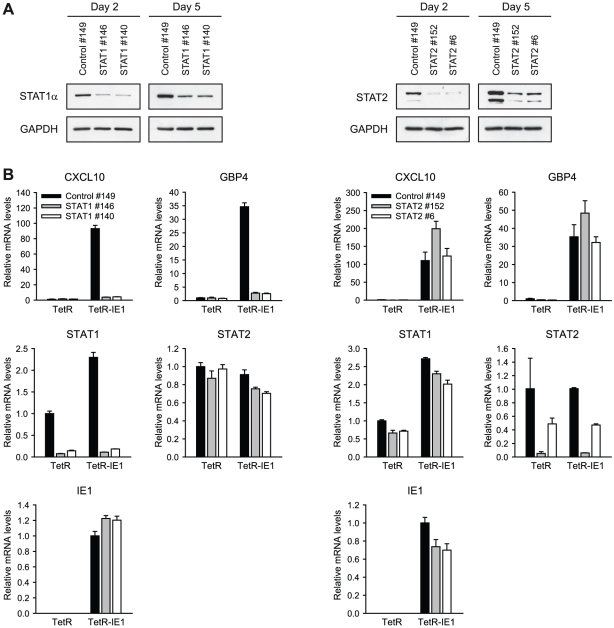
ISG induction by IE1 is dependent on STAT1 but not STAT2. A) Specific reduction in STAT1 (left) and STAT2 (right) protein levels by siRNA-mediated gene silencing. MRC-5 cells were transfected with the indicated siRNA duplexes. Two and five days post transfection, whole cell protein extracts were prepared and subjected to immunoblotting with anti-STAT1α, anti-STAT2, and anti-GAPDH antibodies. B) STAT1 (left) but not STAT2 (right) knock-down abolishes IE1-mediated ISG induction. TetR and TetR-IE1 cells were transfected with the indicated siRNA duplexes. Two days post transfection, cells were treated with doxycycline for 72 h. Relative mRNA expression levels were determined by qRT-PCR with primers specific for the CXCL10, GBP4, IE1, STAT1, and STAT2 genes. Results were normalized to TUBB and mean values with standard deviations from two biological and two technical replicates are shown. CXCL10, GBP4, STAT1, and STAT2 expression is shown in comparison to control siRNA-transfected TetR cells (set to 1). IE1 expression is presented relative to control siRNA-transfected TetR-IE1 cells (set to 1).

To investigate whether ISG activation by IE1 requires the presence of STAT1 and/or STAT2, we employed siRNA-based gene silencing individually targeting the two STAT transcripts. Following transfection of MRC-5, TetR and/or TetR-IE1 cells with two different siRNA duplexes each for STAT1 and STAT2, we monitored endogenous STAT expression by immunoblotting (Figure 7 A) and qRT-PCR (Figure 7 B). An estimated ≥80% selective reduction in STAT1 and STAT2 protein accumulation was observed 2 days following siRNA transfection, and even after 5 days significantly lower protein levels were detected compared to cells transfected with a non-specific control siRNA (Figure 7 A). The knock-down of STAT1 and STAT2 was also evident at the level of mRNA accumulation (86 to 95% for STAT1 and 51 to 95% for STAT2 at day 5 post transfection; Figure 7 B). The knock-down specificity was verified by confirming that STAT1 siRNAs do not significantly reduce STAT2 mRNA levels and *vice versa*. Moreover, none of the STAT-directed siRNAs had any appreciable effect on IE1 expression (Figure 7 B). Again, expression from the CXCL10 and GBP4 genes was strongly up-regulated in doxycycline-treated TetR-IE1 versus TetR cells. However, STAT1 knock-down caused the CXCL10 and GBP4 genes to become almost entirely resistant to IE1-mediated activation in induced TetR-IE1 cells. In contrast, depletion of STAT2 had no negative effect on IE1-dependent ISG induction (Figure 7 B) although it diminished basal and IFN-α-induced type I ISG (OAS1) expression (Supporting Figure S2). These results demonstrate that STAT1, but not STAT2, is an essential mediator of the cellular transcriptional response to IE1 expression and suggest that the viral protein might mediate ISG activation via activation of JAK-STAT signaling.

### IE1-mediated ISG activation requires STAT1 tyrosine phosphorylation

**Figure 8:**
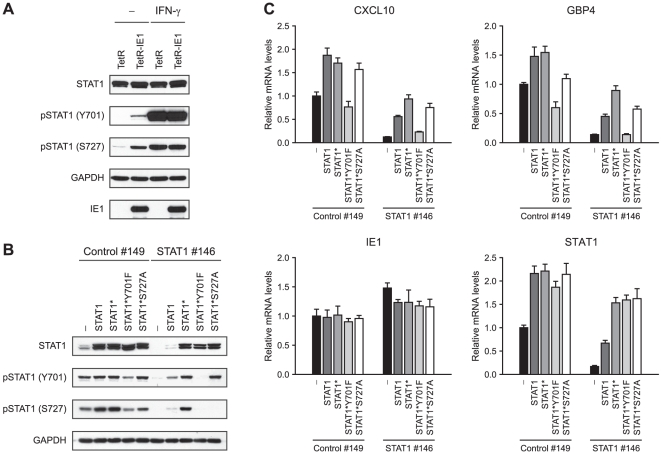
ISG induction by IE1 depends on STAT1 tyrosine phosphorylation. A) IE1 expression leads to increased steady-state levels of Y701- and S727-phosphorylated STAT1. TetR and TetR-IE1 cells were treated for 72 h with doxycycline and for 1 h with solvent (–) or IFN-γ. Whole cell protein extracts were prepared and subjected to immunoblotting with anti-STAT1, anti-pSTAT1 (Y701), anti-pSTAT1 (S727), anti-GAPDH, and anti-IE1 antibodies. B) Verification of knock-down resistance and phosphorylation deficiency of STAT1 variants. TetR-IE1 cells without (–) and with stable expression of ectopic wild-type STAT1 (STAT1), siRNA-resistant wild-type STAT1 (STAT1*), and siRNA-resistant phosphorylation-deficient STAT1 (STAT1*Y701F and STAT1*S727A) were transfected with negative control (#149) or STAT1-specific (#146) siRNA duplexes. Two days post transfection cells were treated for 1 h with IFN-γ. Whole cell protein extracts were prepared and subjected to immunoblotting with anti-STAT1, anti-pSTAT1 (Y701), anti-pSTAT1 (S727), and anti-GAPDH antibodies. C) Ectopic wild-type STAT1 but not phosphorylation-deficient STAT1 mutants efficiently rescue IE1-dependent ISG induction in cells depleted of endogenous STAT1. TetR-IE1 cells without (–) and with stable expression of the indicated ectopic STAT1s were transfected with control (#149) or STAT1-specific (#146) siRNA duplexes. Two days post transfection cells were treated for 72 h with doxycycline. Relative mRNA expression levels were determined by qRT-PCR with primers specific for the CXCL10, GBP4, IE1, and STAT1 genes. Results were normalized to TUBB and mean values with standard deviations from two biological and two technical replicates are shown. Expression is shown in comparison to control siRNA-transfected TetR-IE1 cells without ectopic STAT1 expression (set to 1).

The activation-inactivation cycle of STAT transcription factors entails their transition between different dimer conformations. Unphosphorylated STATs can dimerize in an anti-parallel conformation, whereas tyrosine (Y701) phosphorylation triggers transition to a parallel dimer conformation resulting in increased DNA binding and nuclear retention of STAT1 (reviewed in [29], [30], [97]). In addition, serine (S727) phosphorylation is required for the full transcriptional and biological activity of STAT1 [98]. In order to investigate whether IE1 promotes STAT1 activation, we compared the levels of Y701- and S727-phosphorylated STAT1 in doxycyline-induced TetR and TetR-IE1 cells (Figure 8 A). Total STAT1 steady-state protein levels were very similar in TetR and TetR-IE1 cells. In contrast, Y701-phosphorylated forms of STAT1 were only detectable in the presence of IE1 unless cells were treated with IFN-γ. In addition, IE1 was almost as efficient as IFN-γ in inducing STAT1 S727 phosphorylation. These results strongly suggest that IE1 expression triggers the formation of Y701- and S727-phosphorylated, transcriptionally fully active STAT1 dimers.

To examine whether STAT1 Y701 and/or S727 phosphorylation is an essential step in IE1-mediated ISG activation, we set up a “knock-down/knock-in” system designed to study mutant STAT1 proteins in a context of diminished endogenous wild-type protein levels. We constructed an “siRNA-resistant” STAT1 coding sequence, termed STAT1*, containing two silent nucleotide exchanges in the sequence corresponding to siRNA STAT1 #146 (Figure 7 A). The STAT1* sequence was used as a substrate for further mutagenesis to generate siRNA-resistant constructs encoding mutant STAT1 proteins with conservative amino acid substitutions that preclude tyrosine or serine phosphorylation (Y701F or S727A, respectively; reviewed in [99], [100]). A retroviral gene transfer system based on vector pLHCX was utilized to efficiently express the different STAT1 proteins in TetR-IE1 cells. All STAT1 variants (STAT1*, STAT1*Y701F, and STAT1*S727A) were overexpressed to levels undiscernible from the wild-type protein and mRNA (Figure 8 B–C). In comparison to transfections with a non-specific control siRNA (#149), siRNA #146 severely reduced the levels of endogenous and overexpressed wild-type STAT1 without negatively affecting expression of the siRNA-resistant STAT1 variants or IE1 (Figure 8 B–C). As expected, the Y701F and S727A mutant STAT1 proteins did not undergo tyrosine or serine phosphorylation, respectively, upon stimulation by IFN-γ. Interestingly, while the S727A protein could still be tyrosine-phosphorylated, the Y701F mutant was defective for both tyrosine and serine phosphorylation (Figure 8 B). This observation is in agreement with previous findings showing that IFN-γ-dependent S727 phosphorylation occurs exclusively on Y701-phosphorylated STAT1 [101]. Ectopic expression of wild-type STAT1, STAT1*, and STAT1*S727A but not STAT1*Y701F in addition to the endogenous protein enhanced IE1-mediated activation of CXCL10 and GBP4 transcription. Conversely, siRNA-mediated depletion of endogenous STAT1 strongly reduced this response. Importantly, expression of STAT1* in cells depleted of endogenous STAT1 rescued ISG induction by IE1 almost completely. STAT1*S727A expression also compensated for the lack of endogenous STAT1, although slightly less efficiently compared to STAT1*, whereas STAT1*Y701F was unable to rescue IE1-mediated ISG activation (Figure 8 C).

Thus, although IE1 appears to trigger phosphorylation of STAT1 at both Y701 and S727, only the former modification is required for ISG activation. Nonetheless, STAT1 S727 phosphorylation may augment IE1-dependent gene activation.

### IE1 facilitates STAT1 nuclear accumulation and promoter binding

**Figure 9:**
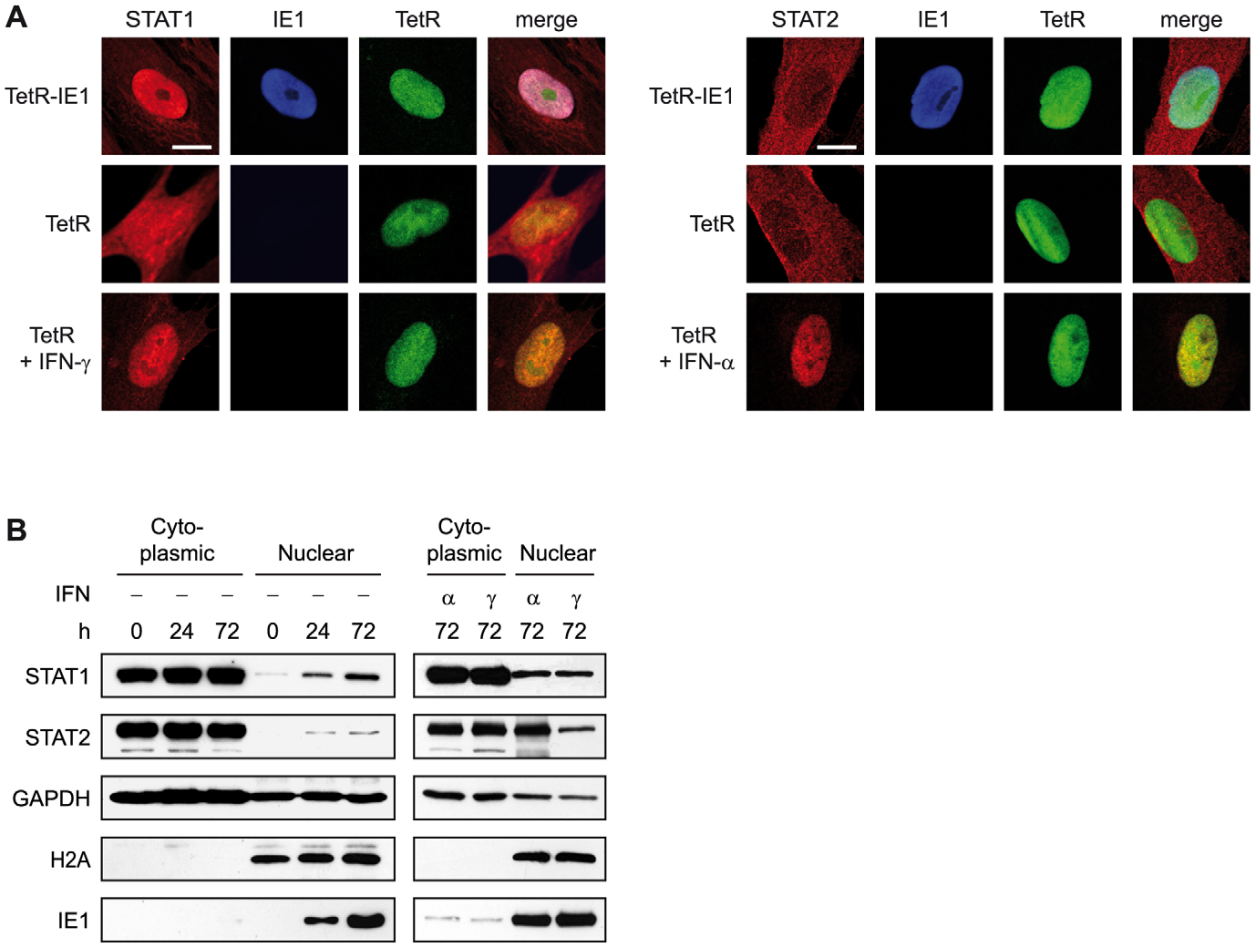
IE1 expression leads to nuclear accumulation of STAT1. A) TetR and TetR-IE1 cells were treated with doxycycline for 72 h. Where indicated, TetR cells were incubated in the presence of IFN-γ or IFN-α for 1 h before samples were fixed with paraformaldehyde and examined by indirect immunofluorescence coupled to confocal microscopy. Samples were simultaneously reacted with rabbit polyclonal antibodies against STAT1 (left) or STAT2 (right) and a mouse monoclonal antibody against IE1, followed by incubation with a rabbit-specific Alexa Fluor 546 conjugate and a mouse-specific Alexa Fluor 633 conjugate. TetRnlsEGFP (TetR) fluorescence is shown to visualize nuclei. Additionally, merge images of STAT, IE1, and TetR signals are presented. Scale bar, 10 µm. B) TetR-IE1 cells were treated with doxycycline for 0 h, 24 h, or 72 h. Cytoplasmic and nuclear extracts were prepared and subjected to immunoblotting with anti-STAT1, anti-STAT2, anti-GAPDH, anti-H2A, and anti-IE1 antibodies. For the right panel, TetR-IE1 cells were treated with IFN-α or IFN-γ for 1 h before fractionation.

Y701 phosphorylation usually causes a cytoplasmic to nuclear shift in steady-state localization and efficient sequence-specific DNA binding of STAT1 dimers (reviewed in [29], [30], [97]). Accordingly, immunofluorescence microscopy revealed that the presence of IE1 strongly promotes nuclear accumulation of STAT1, very similar to what was observed following addition of IFN-γ (Figure 9 A). In contrast, significant amounts of nuclear STAT2 were only detected after treatment of cells with IFN-α but not upon IE1 expression. These results were confirmed by nucleo-cytoplasmic cell fractionation (Figure 9 B). In these assays, IE1 induction for 72 h was as efficient in promoting STAT1 nuclear accumulation as treatment with type I or type II IFNs for 1 h. IFN treatment also strongly induced the nuclear accumulation of STAT2. However, the levels of nuclear STAT2 increased only marginally upon expression of IE1.

**Figure 10:**
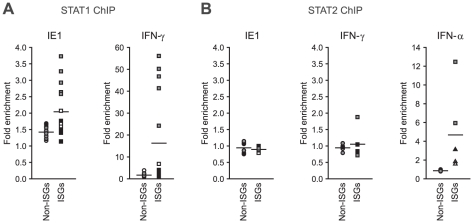
IE1 increases STAT1 occupancy at ISG promoters. TetR and TetR-IE1 cells were treated with doxycycline for 72 h. During the last 30 min of doxycycline treatment TetR cells were incubated in the presence of solvent, IFN-γ or IFN-α. ChIP assays were carried out with polyclonal rabbit antibodies against STAT1 (A) or STAT2 (B). The fraction of immunoprecipitated DNA relative to input DNA was determined by qPCR with primers specific for the non-ISGs GAPDH (white circles), ribosomal protein L30 (RPL30) (black circles), and TUBB (gray circles) as well as for the ISGs GBP4 (white squares), CXCL9 (black squares), TAP1 (gray squares), IFIT2 (white triangles), and OAS1 (black triangles). Mean values of two technical replicates from TetR-IE1 cells (IE1) and from IFN-γ- or IFN-α-treated TetR cells are presented relative to solvent-treated TetR cells (set to 1). Results from five (A) or two (B) independent experiments are shown.

Finally, we asked whether IE1 may direct STAT1 to promoters of type II ISGs. Chromatin immunoprecipitation (ChIP) analyses demonstrated that the viral protein potentiates the recruitment of STAT1 to certain IFN-γ- and IE1-responsive ISG promoters (e.g., TAP1) but not to promoters of several non-ISGs (e.g., GAPDH; Figure 10 A). Moreover, there was a positive correlation between the magnitude of STAT1 chromatin association induced by IE1 and IFN-γ. At the same time, IE1 had no effect on association of STAT2 with these promoters (Figure 10 B). These results are in agreement with the fact that a previous global ChIP-sequencing study has experimentally demonstrated STAT1 association with 14 (56%) out of the 25 IE1-responsive gene promoters identified in this study ([102] and Supporting Table S7). In addition, 22 (88%) of these promoter sequences (all except EDN1, HBG1, and HLA-DRA) carry one or more (up to six) predicted STAT1β binding sites (GAS elements) according to the PROMO tool (version 3.0.2, default settings with 15% maximum matrix dissimilarity rate, http://alggen.lsi.upc.es), which predicts transcription factor binding sites as defined by position weight matrices derived from the TRANSFAC (version 8.3) database [103], [104]. Similar results were obtained with other *in silico* promoter analysis tools (data not shown).

Based on these findings we propose that IE1 activates a subset of ISGs at least in part through increasing the nuclear concentration and sequence-specific DNA binding of phosphorylated STAT1 thereby modulating host gene expression in an unanticipated fashion.

## Discussion

The transcriptional transactivation capacity of the hCMV MIE proteins has been recognized for decades ([72]; reviewed in [2], [40], [41]). For example, it has long been established that the 72-kDa IE1 protein can stimulate transcription from its own promoter-enhancer [36], [105], [106]. IE1 also activates at least a subset of hCMV early promoters therein collaborating with the viral 86-kDa IE2 protein [34], [35], [53], [71], [72], [107], [108], [109]. Furthermore, IE1 or combinations of IE1 and IE2 can stimulate expression from a variety of non-hCMV promoters. In fact, numerous heterologous viral and cellular promoters are responsive to IE1 or combinations of IE1 and IE2 [50], [51], [52], [57], [60], [61], [71], [72], [110], [111], [112], [113], [114], [115], [116], [117]. IE1 may accomplish transcriptional activation via interactions with a diverse set of cellular transcription regulatory proteins thereby acting through multiple DNA elements [50], [51], [52], [54], [55], [56], [57], [58], [59], [105], [106], [109], [110], [111], [112], [113], [117], [118], [119], [120], [121], [122], [123], [124], [125], [126] as well as epigenetic mechanisms including histone acetylation [53], [59], [127]. More recently, IE1 has also been implicated in transcriptional repression [31], [32], [33], [57], [62], [63], [64]. Our own work ([31] and this study, Figure 4 B) and a report by Huh *et al.* (2008) has demonstrated that IE1 can inhibit the hCMV- or IFN-α/β-dependent activation of human ISGs including ISG54, MxA, PKR, and CXCL10. The mechanism of inhibition appears to involve physical interactions of IE1 with the cellular STAT1 and STAT2 proteins that result in diminished DNA binding of the ternary ISGF3 complex to promoters of type I ISGs ultimately interfering with transcriptional activation [31], [32], [33]. Despite this plethora of studies, our understanding of the true transcriptional regulatory capacity of IE1 is still limited. This is mainly due to the fact that IE1-regulated transcription has almost exclusively been studied at the single gene level. Moreover, much of the past work has relied on transfection-based promoter-reporter assays, and IE1-dependent up- or down-regulation of only very few endogenous human genes has been demonstrated so far.

The present work constitutes the first systematic analysis of IE1-specific changes to transcription from the human genome. Importantly, to minimize cellular compensatory effects and to closely mimic the situation during hCMV infection, all experiments were based on short-term (up to 72 h) induction of IE1 expression from its autologous promoter (Figure 1 A–B). Just over 0.1% (25 out of 28,869) of all human transcripts under examination were found to be significantly up-regulated by IE1 under stringent analysis conditions (Table 1). This figure may be unexpected in the light of the reported interactions of IE1 with several ubiquitous transcription factors and its reputation as a “promiscuous” transactivator. However, rather than causing a broad transcriptional host response, IE1-specific gene activation was largely restricted to a subset of ISGs that are primarily responsive to IFN-γ (Table 2, Figure 4 and Supporting Table S4). Thus, IE1 appears to activate certain ISGs (typically type II ISGs) while simultaneously inhibiting the activation of other ISGs (typically type I ISGs). Importantly, more than half (at least 14 out of the 25) IE1-activated genes identified in this study were previously shown to be induced during hCMV infection of fibroblasts and/or other human cell types (Table 3). This strongly suggests that many if not all IE1-specific transcriptional changes observed in our expression model may be relevant to viral infection. On the other hand, our preliminary results indicate that the conditional replication defect of IE1 knock-out viruses in human fibroblasts [35], [36] may not result from an inability to initiate an IFN-γ-like response (data not shown). In fact, additional viral gene products are known or expected to contribute to ISG activation during hCMV infection (reviewed in [26], [27]) and may compensate for IE1 in this respect, at least during productive infection of fibroblasts.

**Table 3:** IE1-activated human genes shown to be induced during hCMV infection.

Gene symbol	mRNA^1^	Protein^2^	References
IFI44L	+	−	[162]
GBP1	+	−	[12], [18], [161], [224]
GBP2	+	−	[13], [15], [18], [161], [224]
GBP4	+	−	This work^3^
GBP5	−	−	
CTSS	+	+	[4], [24]
TNFSF18	−	−	
TNFSF4	−	−	
SERTAD4	−	−	
HES1	+	−	[18]
CXCL9	+	+	[161], [165] and this work^3^
CXCL10	+	+	[4], [18], [24], [160], [161], [162], [163], [164] and this work^3^
CXCL11	+	+	[4], [18], [161], [162] and this work^3^
IRF1	+	−	[12], [15], [16], [17],[18],[162]
EDN1	−	−	
HLA-DRA	+	−	[14]
TAP1	+	−	[14], [18], [162]
IDO1	+	−	[15], [18], [161] and this work^3^
CD274	−	−	
CCDC3	−	−	
IFIT2	+	−	[12], [15], [16], [18], [96], [161], [162], [194], [225], [226], [227], [228]
IFIT3	+	−	[15], [162], [194], [224], [228]
ANKRD1	−	−	
HBG1	−	−	
CCL11	−	−	

1 Up-regulated at the level of mRNA accumulation.

2 Up-regulated at the level of protein accumulation and/or secretion.

3 Up-regulated at mRNA level by TNwt infection of MRC-5 cells (data not shown).

+ = reported to be up-regulated by hCMV; − = not reported to be up-regulated by hCMV.

In addition to being distinctively responsive to IFN-γ, most IE1-activated genes appear to share similar kinetics of induction (Table 1 and Figure 3), and many cluster in certain genomic locations (Table 2) suggesting a common underlying mechanism of activation. Specific siRNA-mediated STAT1 (but not STAT2) knock-down inhibited IE1-dependent activation of several target ISGs almost completely (Figure 7 A). Conversely, STAT1 overexpression proved to enhance ISG activation in IE1 expressing cells (Figure 8 C). Moreover, defective IE1-activated ISG transcription in cells depleted of endogenous STAT1 was efficiently rescued by ectopic STAT1 expression (Figure 8 C). These results demonstrate that the STAT1 protein is a critical mediator of the cellular transcriptional response to IE1. Moreover, this response appears to strictly depend on the Y701-phosphorylated form of STAT1 which is induced by IE1 expression (Figure 8). Although recent work has shown that some STAT1 functions are executed by the non-phosphorylated protein (reviewed in [97], [99], [100]), it is the Y701-phosphorylated form that preferentially accumulates in the nucleus and binds to DNA with high affinity (reviewed in [29], [30]) providing a mechanism for IE1-dependent ISG activation. IE1 also induces S727 phosphorylation of STAT1 (Figure 8 A), but this modification is dispensable merely serving an augmenting function in ISG activation triggered by the viral protein (Figure 8 C). Phosphorylation of S727 is thought to be required for the full transcriptional activity of STAT1 by recruiting histone acetyltransferase activity [98], [128], [129]. Interestingly, the hCMV IE1 protein can promote histone acetylation [53] suggesting it might compensate for S727 phosphorylation by binding to DNA-associated STAT1.

Our prior work has shown that IE1 physically interacts with STAT1 during hCMV infection and *in vitro*, and the two proteins co-localize in the nuclei of transfected cells treated with IFN-α [31]. The results of Figure 9 extend these observations by demonstrating that the viral protein facilitates nuclear accumulation and DNA binding of STAT1 in the absence of IFNs. The STATs were initially described as cytoplasmic proteins that enter the nucleus only in the presence of cytokines. However, it has now been established that STATs constantly shuttle between nucleus and cytoplasm irrespective of cytokine stimulation (reviewed in [97], [130], [131]). Thus, complex formation between nuclear resident IE1 and STAT1 passing through the nucleus may be sufficient to impair STAT1 export to the cytoplasm resulting in nuclear retention and increased DNA binding of the cellular protein. In this scenario, IE1 may increase the levels of Y701-phosphorylated STAT1 by interfering with nuclear dephosphorylation of the cellular protein. In fact, DNA binding was shown to protect STAT1 from dephosphorylation, which normally occurs at a step preceding export to the cytoplasm [132], [133]. This one-step “nuclear shortcut” model assumes that small amounts of Y701-phosphorylated STAT1 enter the nucleus in the absence of IFNs and any potential IE1-induced mediators of STAT1 activation. Conceivably, human fibroblasts (TetR cells) may constitutively release small amounts of soluble inducers (e.g., certain growth factors; see below) that maintain residual levels of activated STAT1 undetectable by immunoblotting (Figure 8 A). Moreover, we cannot rule out that the fetal calf serum used for cell culture media may contain factors causing a limited number of STAT1 molecules to undergo Y701 phosphorylation. In contrast, increased S727 phosphorylation in the presence of IE1 may result from higher levels of DNA-targeted STAT1, as this modification is preferentially or exclusively incorporated into the nuclear chromatin-associated cellular protein, at least during the normal IFN-γ response [101].

Alternatively, IE1 may actively induce STAT1 Y701 phosphorylation thereby promoting nuclear import of STAT1 dimers. This phosphorylation event is typically mediated by cytoplasmic JAK family kinases upon ligand-mediated activation of IFN receptors. However, our results demonstrate that IE1 does not induce the expression of human IFN genes, and we found no evidence for IFN-γ or IFN-β secretion from IE1 expressing cells (Supporting Table S5, Figure 6 and data not shown). Moreover, our transwell and media transfer experiments indicate that cytokines or other soluble mediators that may constitute a hypothetical IE1 “secretome” are not sufficient to stimulate ISG expression (Figure 5 and data not shown). However, this does not rule out the possibility that IE1 may cooperate with one or more soluble factors to trigger the observed transcriptional response. In fact, 80% of all IE1 target genes were not found activated within the first 24 h after induction of IE1 expression despite the fact that the viral protein had reached almost peak levels by this time (Figure 1 B and Table 1). Instead, up-regulation typically started at 48 h and increased until at least 72 h following IE1 expression (Table 1 and Figure 3 A). This timing of induction is compatible with a two-step model in which IE1 first initiates *de novo* synthesis and secretion of an unidentified cellular gene product required to trigger STAT1 Y701 phosphorylation (step 1). Besides IFNs, STAT1 signaling can be induced by several interleukins (e.g., IL-6) some of which are known to be up-regulated by IE1 [58], [60], [61], [110]. However, STAT1 Y701 phosphorylation can also occur independently of cytokines (reviewed in [134]). In fact, growth factors including the epidermal growth factor and certain hormones are also able to induce STAT1 Y701 phosphorylation [135], [136], [137], [138], [139]. In addition, tumor necrosis factor (TNF) has been shown to signal through activated STAT1 [140] raising the intriguing possibility that the soluble protein products of TNFSF4 and/or TNFSF18, two TNF family members belonging to the few genes already activated by 24 h following IE1 induction (Table 1), may be involved in IE1-mediated Y701 phosphorylation of STAT1. However, activation of one or more of these IFN-independent pathways may not produce enough activated nuclear STAT1 to trigger efficient ISG expression and may therefore be required but not sufficient for IE1-mediated gene induction. In accordance with this possibility, the levels of Y701-phosphorylated STAT1 were much higher in IFN-γ-treated as compared to IE1 expressing cells (Figure 8 A). Thus, on top of low level Y701 phosphorylation, IE1-dependent nuclear retention of STAT1 through complex formation between the viral and cellular protein (as outlined for the one-step model; see above) may be necessary in order to elicit a significant transcriptional response (step 2).

Although activated STAT1 is clearly a key mediator of IE1-dependent ISG induction, additional factors may be involved. In fact, not all known STAT1-activated human genes seem to be included in the IE1-specific transcriptome implying that additional gene products likely contribute to target specificity. One of the candidate co-factors that has been repeatedly linked to IE1 function is NFκB. In fact, IE1 was shown to activate the NFκB p65 (RelA) and RelB promoters [55], [112], [113], [121], to facilitate expression of the NFκB RelB subunit and/or NFκB post-translational activation [58], [113], [119], [121], and to activate transcription through NFκB binding sites [58], [105], [106], [113], [119], [126]. At the same time, NFκB has been implicated in IFN-γ-induced activation of a subset of ISGs including CXCL10 and GBP2 ([141], [142], [143], [144], [145]; reviewed in [146], [147]). However, we did not observe nuclear translocation of NFκB following induction of IE1 in TetR-IE1 cells. Moreover, siRNA-mediated knock-down of NFκB p65 had no significant impact on IE1-activated CXCL10 and GBP4 expression in these cells (data not shown). These observations indicate that the transcriptional response to IE1 is largely independent of NFκB, at least within our experimental setup. IRF1 is another transcription factor that contributes to the activation of certain ISGs including CTSS, GBP2, and TAP1 ([128], [148], [149], [150]; reviewed in [80], [81], [82]). IRF1 might enhance IE1-mediated ISG activation, especially since its mRNA is up-regulated by expression of the viral protein (Table 1 and Figure 4 A).

A key feature of the IE1 protein appears to be its ability to target to and disrupt subnuclear multi-protein structures known as PML bodies or ND10 during the early phase of hCMV infection and upon ectopic expression [42], [43], [44]. The mechanism of IE1-dependent ND10 disruption most likely involves binding to the PML protein, a major constituent of ND10 [54]. We have not specifically investigated the role of PML in IE1-mediated gene induction. Nonetheless, our results are compatible with the possibility that ND10 disruption is required for the transcriptional response to IE1 since the nuclear structures were confirmed to be disintegrated at both post-induction time points (24 h and 72 h) of our microarray analysis (data not shown). Although the exact function of ND10 remains unclear, the structures have been implicated in a variety of processes including inflammation [151] and anti-viral defense (reviewed in [45], [46], [47], [48]). Besides a proposed role of ND10 in viral gene expression, they may also function in transcriptional regulation of certain cellular genes. Several examples of selective associations between ND10 and genes or chromosomal loci, especially regions of high transcription activity and/or gene density, have been reported (reviewed in [152]). For example, immunofluorescent *in situ* hybridization analyses demonstrated that the major histocompatibility (MHC) class I gene cluster on chromosome 6 (6p21) is non-randomly associated with ND10 in human fibroblasts [153]. Transcriptional activation in the presence of IFN-γ correlates with the relocalization of this locus to the exterior of the chromosome 6 territory in a process that appears to involve DNA binding of Y701-phosphorylated STAT1, changes in chromatin loop architecture, and histone hyperacetylation [154], [155], [156]. Interestingly, many IE1-activated genes cluster in certain genomic locations (Table 2). This includes the HLA-DRA and TAP1 genes located within the ND10-associated MHC locus at 6p21. Together these observations raise the intriguing possibility that, through a combination of PML disruption and STAT1 activation, IE1 might cause higher order chromatin remodeling of entire chromosomal loci resulting in transcriptional activation.

One of the most surprising findings of the present study concerns the fact that most IE1-induced cellular genes are generally associated with stimulatory rather than inhibitory effects on immune function and inflammation (Table 1, Figure 2 and Supporting Tables S1, S2). It has been proposed that certain inflammatory and innate defense mechanisms launched by the host to limit hCMV replication may actually facilitate viral dissemination, for example by increasing target cell availability and/or by creating an environment conducive to virus reactivation (coined “no pain, no gain” by Mocarski [157]). Thus, it is plausible that hCMV not just attenuates host immunity through the numerous immune evasion mechanisms ascribed to this virus (reviewed in [158]), but rather aims at counterbalancing the effects of the innate and inflammatory response in restricting and facilitating viral replication. This strategy may be crucial in allowing for what has been termed “mutually assured survival” of both virus and host [159].

The functional group of IE1-induced pro-inflammatory proteins potentially involved in viral target cell recruitment is best represented by the chemokines CXCL9, CXCL10, and CXCL11. All three proteins are not only induced by IE1 (Table 1 and Figures 3–7) but also during hCMV infection of various cell types, and they represent major constituents of the viral secretome ([4], [18], [24], [160], [161], [162], [163], [164], [165] and Table 3). By binding to a common receptor, termed CXCR3, the three chemokines have the ability to attract subsets of circulating leukocytes to sites of infection and/or inflammation (reviewed in [74], [75]). Although CXCR3 is preferentially expressed on activated T helper 1 cells, the receptor protein is also present on many other cell types including CD34+ hematopoietic progenitors [166] which are preferential sites of hCMV latency [167], [168], [169], [170], [171], [172]. CXCR3 and its ligands have been implicated in a large variety of inflammatory and immune disorders (reviewed in [74], [75]). For example, cells expressing CXCR3 are found at high numbers in biopsies taken from patients experiencing organ transplant dysfunction and/or rejection [173], [174], [175], [176], [177], [178], [179], [180], [181]. Moreover, CXCL9 [175], [176], [177], [179], [180], CXCL10 [173], [174], [175], [176], [177], [179], [180], and CXCL11 [175], [176], [177], [178], [179], [180], [181] mRNA and protein levels are increased in tissues of organs undergoing rejection. Importantly, the levels of CXCR3-positive cells and CXCR3 ligand mRNA in the biopsy samples frequently correlate with the grade of graft rejection [174], [176], [177], [178], [180] suggesting a causative role of this pathway. Up-regulation of CXCL10 and other chemokines also correlated with transplant vascular sclerosis and chronic rejection in an rCMV cardiac allograft infection model [4], [182], [183]. In addition to CXCL9, CXCL10, and CXCL11, IE1 also up-regulates expression of CCL11 (Table 1), another CXCR3-interacting chemokine [184]. Through activation of the CXCR3 axis, IE1 might contribute to hCMV dissemination and pathogenesis in unexpected ways.

The IE1 protein has long been suspected to be a key player in the events leading to reactivation from hCMV latency although this view has recently been challenged by functional analysis of the mCMV and rCMV IE1 orthologs in mouse and rat models of infection, respectively [37], [185]. Nonetheless, inflammatory (including allogeneic) immune responses are believed to be efficient stimuli for hCMV reactivation. In fact, stimulation of latently infected monocytes or myeloid progenitor cells with pro-inflammatory cytokines including IFN-γ can reactivate viral replication ([186], [187], [188], [189]; reviewed in [190], [191], [192]). IFN-γ may aid hCMV reactivation by affecting cellular differentiation ([193]; reviewed in [28], [190], [191], [192]) and/or by activating transcription through GAS-like elements present in the viral MIE promoter-enhancer [194]. These GAS-like elements were shown to be required for efficient hCMV transcription and replication, at least after low multiplicity infection, and IFNs enhanced MIE gene expression [194]. Conceivably, the IE1 protein may phenocopy the effect of IFN-γ in activating both cellular ISGs and the viral MIE promoter thereby facilitating viral reactivation. Conversely, along the lines of the “immune sensing hypothesis of latency control” proposed by Reddehase and colleagues [195], episodes of IE1 expression may promote maintenance of viral latency not only through providing antigenic peptides (reviewed in [196]) but also by concomitantly activating critical immune effector functions including antigen transport (TAP1), processing (CTSS) and presentation (HLA-DRA) as well as immune cell recruitment (CXCL9, CXCL10, CXCL11, CCL11; see above) and co-stimulation (TNFSF4, TNFSF18 and CD274).

Current anti-hCMV strategies are directed against viral DNA replication, but sometimes fail to halt disease. This may be due to virus-induced “side effects” that are not correlated to production of virus particles and lysis of host cells. In fact, in hCMV pneumonitis and retinitis, disease symptoms were repeatedly found in the absence of replicating virus or viral cytopathogenicity [197], [198]. Similarly, in mouse models of viral pneumonitis mCMV replication *per se* was not sufficient to cause disease [197], [199], [200]. Conversely, mCMV disease could be triggered immunologically without inducing viral replication [201]. Here we have shown that out of >160 different hCMV gene products, a single protein (IE1) is sufficient to alter the expression of human genes with strong pro-inflammatory and immune stimulatory potential without the requirement for virus replication. The present work supports the idea that the hCMV MIE gene and specifically the IE1 protein may play a direct and predominant role in viral immunopathogenesis and inflammatory disease [202], [203], [204], [205]. Thus, the IE1 protein should be considered a prime target for the development of improved prevention and treatment options directed against hCMV.

## Materials and Methods

### Plasmids

The pMD2.G and psPAX2 packaging vectors for recombinant lentivirus production were obtained from Addgene (http://www.addgene.org; plasmids 12259 and 12260, respectively). Plasmids pLKOneo.CMV.EGFPnlsTetR, pLKO.DCMV.TetO.cICP0, and pCMV.TetO.cICP0 were kindly provided by Roger Everett (Glasgow, UK). pLKOneo.CMV.EGFPnlsTetR contains the complete hCMV MIE promoter upstream of a sequence encoding EGFP fused to an NLS and TetR [68], [69], [70]. In the pLKO.1puro derivative pLKO.DCMV.TetO.cICP0, expression of the herpes simplex virus type 1 infected cell protein 0 cDNA (cICP0) is under the control of a tandem TetO sequence located downstream of a truncated version of the hCMV MIE promoter (DCMV) [69], [70]. To generate pLKO.DCMV.TetO.cIE1, the IE1 cDNA of the hCMV Towne strain was PCR-amplified from pEGFP-IE1 [71] with upstream primer #483 containing a *Hind*III site and downstream primer #484 containing an *Eco*RI site (the sequences of all primers used in this study are listed in Supporting Table S8). The IE1 sequence was subcloned into the *Hind*III and *Eco*RI sites of pCMV.TetO.cICP0. The *Nde*I-*Eco*RI fragment of the resulting plasmid pCMV.TetO.IE1 was verified by sequencing and used to replace the ICP0 cDNA in pLKO.DCMV.TetO.cICP0 thereby generating plasmid pLKO.DCMV.TetO.cIE1.

QuikChange site-directed mutagenesis of plasmid pRc/CMV-hSTAT1p91 (kindly provided by Christian Schindler, New York, USA) with oligonucleotides #660 and #661 resulted in pCMV-STAT1* encoding a STAT1 variant mRNA resistant to silencing by the STAT1-specific siRNA duplex #146 (the sequences of all siRNAs used in this study are listed in Supporting Table S9). The plasmids pCMV-STAT1*Y701F and pCMV-STAT1*S727A were generated by QuikChange mutagenesis of pCMV-STAT1* with primer pairs #662/#663 and #664/#665, respectively. *Bam*HI-*Eco*RV fragments of pRc/CMV-hSTAT1p91, pCMV-STAT1*, pCMV-STAT1*Y701F, and pCMV-STAT1*S727A were treated with Klenow fragment and ligated to the *Hpa*I-digested, dephosphorylated retroviral vector pLHCX (Clontech, no. 631511) resulting in plasmids pLHCX-STAT1, pLHCX-STAT1*, pLHCX-STAT1*Y701F, and pLHCX-STAT1*S727A, respectively. The correct orientations and nucleotide sequences of the inserted STAT1 cDNAs were verified by sequencing.

### Cells and retroviruses

Human MRC-5 embryonic lung fibroblasts (Sigma-Aldrich, no. 05011802), the human p53-negative non-small cell lung carcinoma cell line H1299 (ATCC, no. CRL-5803 [206]), and Phoenix-Ampho retrovirus packaging cells (from Garry Nolan, Stanford, USA [207]) were maintained in Dulbecco's Modified Eagle's Medium supplemented with 10% fetal calf serum, 100 units/ml penicillin, and 100 µg/ml streptomycin. All cultures were regularly screened for mycoplasma contamination using the PCR Mycoplasma Test Kit II from PromoKine. Where applicable, cells were treated with 1,000 U/ml recombinant human IFN-α A/D (R&D Systems, no. 11200), 10 ng/ml recombinant human IFN-β 1a (Biomol, no. 86421), or 10 ng/ml recombinant human IFN-γ (R&D Systems, no. 285-IF) for various durations. Neutralizing goat antibodies to human IFN-β (no. AF814) or IFN-γ (no. AF-285-NA) and normal goat IgG (no. AB-108-C) were purchased from R&D Systems and used at concentrations of 1 µg/ml (anti-IFN-β) or 2 µg/ml (anti-IFN-γ, normal IgG). Transwell assays were performed in tissue-culture-treated 100-mm plates with polycarbonate membrane and 0.4 µm pore size (Corning, no. 3419).

During the week prior to transfection, Phoenix-Ampho cells were grown in medium containing hygromycin (300 µg/ml) and diphtheria toxin (1 µg/ml). Production of replication-deficient retroviral particles, retrovirus infections, and selection of stable cell lines were performed according to the pLKO.1 protocol available on the Addgene website (http://www.addgene.org/pgvec1?f=c&cmd=showcol&colid=170&page=2) with minor modifications. Retroviral particles were generated by transient transfection of H1299 cells (pLKO-based vectors) or Phoenix-Ampho cells (pLHCX-based vectors) using the calcium phosphate co-precipitation technique [208]. Recombinant viruses were collected 36 h and 60 h after transfection, and were used for transduction of target cells by two subsequent 16 h incubations. To generate TetR cells, MRC-5 fibroblasts at population doubling 19 were infected with pLKOneo.CMV.EGFPnlsTetR-derived lentiviruses and selected with G418 (0.2 mg/ml). To generate TetR-IE1 cells, TetR cells were transduced by pLKO.DCMV.TetO.cIE1-derived lentiviruses and selected with puromycin (1 µg/ml). Cells with high level EGFPnlsTetR expression (and low IE1 background) were enriched by fluorescence-activated cell sorting in a FACSCanto II flow cytometer (BD Biosciences). TetR cells were maintained in medium containing G418 (0.1 mg/ml), while TetR-IE1 cells were cultured in the presence of both G418 (0.1 mg/ml) and puromycin (0.5 µg/ml). To induce IE1 expression, cells were treated with doxycycline (Clontech, no. 631311) at a final concentration of 1 µg/ml. To generate TetR-IE1 cells with stable expression of ectopic STAT1 proteins, uninduced TetR-IE1 cells were transduced with pLHCX-derived retroviruses encoding STAT1, STAT1*, STAT1*Y701F, or STAT1*S727A.

### hCMV mutagenesis and infection

The EGFP-expressing wild-type Towne strain (TNwt) of hCMV was derived from an infectious BAC clone (T-BACwt [209]) of the viral genome. Allelic exchange to generate IE1-deficient viruses (TN*dl*IE1) and corresponding “revertants” (TN*rv*IE1) utilized the following derivatives of transfer plasmid pGS284 [210]: pGS284-TNIE1*kanlac*Z, pGS284-TNMIE*dl*IE1, pGS248-TNMIE, and pGS284-TNMIE*rv*IE1. Plasmid pGS284-TNIE1*kanlac*Z contains the kanamycin resistance gene (*kan*) and the *lac*Z gene cloned between sequences flanking the IE1-specific exon four of the hCMV TN MIE transcription unit. The ∼1000-bp flanking sequences were obtained by PCR amplification using primers #136 and #137 (downstream flanking sequence) or #139 and #140 (upstream flanking sequence; for PCR primer sequences, see Supporting Table S8) and T-BACwt as template. The amplified downstream flanking sequence was cloned into pGS284 via *Bgl*II and *Not*I sites present in both the PCR primers and target vector sequences. Following addition of adenosine nucleotide overhangs to the 3′-ends of the PCR product, the upstream flanking sequence was first subcloned into vector pCR4-TOPO (Invitrogen) and subsequently inserted via *Not*I sites into pGS284 carrying the downstream flanking sequence. The *kanlac*Z expression cassette was released from plasmid YD-C54 [211] and cloned into the *Pac*I sites (introduced through PCR primers #137 and #139) located between the hCMV flanking sequences in the pGS284 derivative described above. Plasmid pGS284-TNMIE*dl*IE1 contains an MIE fragment lacking 1,413 bp between the *Acc*I sites upstream and downstream of exon four. The exon four-deleted MIE fragment was obtained from T-BACwt by overlap extension PCR as previously described [212]. The primer pairs used for PCR mutagenesis were #348/#349 (upstream fragment), #350/#351 (downstream fragment), and #348/#351 (complete fragment). The final PCR product was cloned via *Bgl*II and *Not*I sites into pGS284. For the construction of pGS248-TNMIE (previously termed pGS248-MIE; [33]), a ∼3000-bp sequence of the MIE region was amplified by PCR using template T-BACwt and primers #155 and #156. After phosphorylation, the PCR product was first inserted into the *Sma*I site of pUC18 and then excised from this vector via *Fse*I and *Not*I sites. The *Fse*I-*Not*I fragment was subsequently cloned into the same sites of pGS284-TNMIE*dl*IE1 thereby repairing the exon four deletion in this plasmid to generate pGS284-TNMIE*rv*IE1. DNA sequence analysis was completed on all hCMV-specific PCR amplification products to confirm their integrity. Allelic exchange was performed through homologous recombination in *Escherichia coli* strain GS500 as previously described [33], [210], [211]. First, the BAC pTNIE1*kanlac*Z was generated by recombination of T-BACwt with pGS284-TNIE1*kanlac*Z followed by selection for kanamycin resistance and LacZ expression. After that, the BACs pTN*dl*IE1 and pTN*rv*IE1 were made through recombination of pTNIE1*kanlac*Z with pGS284-TNMIE*dl*IE1 and pGS284-TNMIE*rv*IE1, respectively, followed by selection for the loss of kanamycin resistance and LacZ expression. The BAC constructs were analyzed by *Eco*RI digestion. The BACs pTN*dl*IE1 and pTN*rv*IE1 were used for electroporation of MRC-5 cells to reconstitute viruses TN*dl*IE1 and TN*rv*IE1, respectively, as has been described previously [211]. Cell- and serum-free virus stocks were produced upon BAC transfection of MRC-5 fibroblasts (TNwt and TN*rv*IE1) or TetR-IE1 cells (TN*dl*IE1), and the titers of the wild-type TN and revertant preparations were determined by standard plaque assay on MRC-5 cells. Titration of TN*dl*IE1 stocks was performed by quantification of intracellular genome equivalents [33]. Multistep replication analysis of recombinant viruses on TetR and TetR-IE1 cells has been described previously [33].

### GeneChip analysis

For global transcriptome analysis, 1.9×10^6^ TetR or TetR-IE1 cells of the same passage number were seeded on 10-cm dishes. When cells reached confluency (three days after plating), the medium was replaced, and cells were growth-arrested by maintaining them in the same medium for seven days before they were collected for transcriptome analysis. During the last 72 h or 24 h prior to collection, cultures were treated with doxycycline at a final concentration of 1 µg/ml or were left untreated. Total RNA was isolated using TRIzol reagent (Invitrogen) and Phase Lock Gel Heavy (Eppendorf) according to the manufacturers' instructions. A second purification step with on-column DNase digestion was performed on the isolated RNA using the RNeasy Mini Kit from Qiagen. All subsequent steps were performed at the Kompetenzzentrum für Fluoreszente Bioanalytik (Regensburg, Germany). Total RNA (100 ng) was labeled using reagents and protocols specified in the Affymetrix GeneChip Whole Transcript (WT) Sense Target Labeling Assay Manual (P/N 701880 Rev. 4). Quantity and quality of starting total RNA, cRNA, and single-stranded cDNA were assessed in a NanoDrop spectrophotometer (Thermo Fisher Scientific) and a 2100 Bioanalyzer (Agilent Technologies), respectively. Samples were hybridized to Affymetrix Human Gene 1.0 ST Arrays which interrogate 28,869 well-annotated genes and cover >99% of sequences present in the RefSeq database (National Center for Biotechnology Information). We probed a total of 18 microarrays, which allowed us to monitor three biological replicates for each experimental condition (TetR and TetR-IE1 cells without and with 24 h and 72 h of doxycycline treatment). For creation of the summarized probe intensity signals, the Robust Multi-Array Average algorithm [213] was used. Files generated by the Affymetrix GeneChip Operating 1.4 and Expression Console 1.1 software have been deposited in Gene Expression Omnibus (GEO, National Center for Biotechnology Information [214]) and are accessible through GEO Series accession number GSE24434 (http://www.ncbi.nlm.nih.gov/geo/query/acc.cgi?acc=GSE24434).

### qRT-PCR

In order to determine steady-state mRNA levels by qRT-PCR, total RNA was isolated from 3 to 4×10^5^ fibroblasts using Qiagen's RNeasy Mini Kit and RNase-Free DNase Set according to the manufacturer's instructions. First-strand cDNA was synthesized using SuperScript III and Oligo(dT)_20_ primers (Invitrogen) starting from 2 µg of total RNA. Unless otherwise noted, first-strand cDNA was diluted 10-fold with sterile ultrapure water, and 5 µl were used to template 20-µl real-time PCRs performed in a Roche LightCycler 1.5 [33]. The instrument was operated with a ramp rate of 20°C per sec using the following protocol: pre-incubation cycle (95°C for 10 min, analysis mode: none), 40 to 50 amplification cycles with single fluorescence measurement at the end of the extension step (denaturation at 95°C for 10 sec, primer-dependent annealing at 66 to 56°C for 10 sec, primer-dependent extension at 72°C for 8 to 10 sec, analysis mode: quantification), melting curve cycle with continuous data acquisition during the melting step (denaturation at 95°C for 0 sec, annealing at 65°C for 60 sec, melting at 95°C for 0 sec with a ramp rate of 0.1°C/sec, analysis mode: melting curves), cooling cycle (40°C for 30 sec, analysis mode: none). The PCR mix was composed of 9 µl PCR grade water, 1 µl forward primer solution (10 µM), 1 µl reverse primer solution (10 µM), and 4 µl 5× concentrated Master Mix from the LightCycler FastStart DNA Master^PLUS^ SYBR Green I kit. The sequences of the high pressure liquid chromatography-purified PCR primers are listed in Supporting Table S8. All samples were quantified at least in duplicate, and each analysis included positive, minus-RT, and non-templated controls. The second derivate maximum method with arithmetic baseline adjustment (LightCycler Software 3.5) was used to determine quantification cycle (Cq) values. Cq values were further validated by ensuring they meet the following criteria: (i) corresponding melting peaks of the generated PCR products, calculated using the polynomial method with digital filters enabled, had to match the single peak of the positive control sample, (ii) standard deviations of Cq values from technical replicates had to be below 0.33, (iii) Cq values had to be significantly different from minus-RT controls (Cq
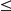
Cq_-RT_-1), and (iv) Cq values had to be within the linear quantification range. The linear quantification range was individually determined for each primer pair by generating a standard curve with serial dilutions of first-strand cDNA from the sample with the highest expression level. PCR efficiency (*E*) was calculated from the slope of the standard curve according to equation (1):
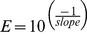
(1)The relative expression ratio (*R*) of the target (*trgt*) and reference (*ref*) gene in an experimental (*eptl*) versus control (*ctrl*) sample was calculated using the efficiency-corrected model shown in equation (2):
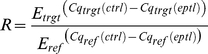
(2)


Control samples of all experiments had reference and target gene expression levels well above the limits of detection. The tubulin-β gene (TUBB) was chosen as a reference, because (i) expression levels did not change upon IE1 induction, IFN treatment, siRNA transfection, or hCMV infection, (ii) it allowed for RNA-specific detection with no spurious product generation in minus-RT controls, and (iii) it exhibited similar expression levels compared to the target genes under investigation, which were generally expressed at levels lower than TUBB in the absence and at similar or higher levels relative to TUBB in the presence of IE1 expression, IFN treatment, or hCMV infection.

### Chemokine quantification

CXCL9, CXCL10, and CXCL11 chemokine concentrations in cell culture supernatants were determined using commercially available colorimetric sandwich enzyme immunoassay kits (Quantikine Immunoassays no. DCX900, DIP100, and DCX110 from R&D Systems) following the manufacturer's instructions.

### RNA interference

The sequences of siRNA duplexes used for mRNA knock-down experiments are listed in Supporting Table S9. They were introduced into cells at 30 nM final concentration using the Lipofectamine RNAiMAX Reagent (Invitrogen) following the manufacturer's instructions. Briefly, exponentially growing cells were seeded either in 12-well dishes at 2.5×10^5^ cells/well for RNA analyses or in 6-well dishes at 5×10^5^ cells/well for protein analyses. Transfections were performed in Opti-MEM I Reduced Serum Medium (Invitrogen) with 2 µl or 5 µl of RNAiMAX Reagent for 12- or 6-wells, respectively.

### Subcellular fractionation, immunoblotting, and microscopy

Cells (3.8×10^6^) on 10-cm dishes were collected with trypsin/EDTA and then centrifuged for 5 min at 500× g and 4°C. Supernatants were removed and cells resuspended in 100 µl CSK buffer (10 mM PIPES [pH 6.8], 300 mM sucrose, 100 mM NaCl, 3 mM MgCl_2_, 1 mM EDTA, 0.1% (v/v) Igepal CA-630) with freshly added protease and phosphatase inhibitor cocktails. Lysates were centrifuged for 1 min at 1,300× g and 4°C, and the supernatants (cytoplasmic extracts) were transferred to clean pre-chilled tubes and combined with one volume of 2× protein sample buffer (100 mM Tris-HCl [pH 6.8], 4% (w/v) SDS, 20% (v/v) glycerol, 200 mM β-mercaptoethanol, 0.1% (w/v) bromophenol blue). The insoluble (pellet) fractions containing nuclei were washed once with 500 µl CSK buffer before they were suspended in 200 µl 2× protein sample buffer and sonified in a Bioruptor (Diagenode; “H” setting; 30 sec on-off interval) for 15 min. Samples were centrifuged for 10 min at 20,000× g and 4°C, and the supernatants (nuclear extracts) were transferred to clean pre-chilled tubes. Cytosolic and nuclear extracts were heated to 95°C for 5 min before immunoblot analysis. Generation of whole cell extracts, sodium dodecyl sulfate-polyacrylamide gel electrophoresis, immunoblotting, and (immuno)fluorescence microscopy were performed according to previously published protocols [33], [53], [215]. Immunodetection employed primary mono- or polyclonal antibodies directed against hCMV IE1 (1B12; [216]) or human GAPDH (Abcam, no. ab9485), histone H2A (Abcam, no. ab13923), STAT1 (no. sc-464 for immunoblotting and no. sc-346 for immunofluorescence, both from Santa Cruz), STAT1α (Santa Cruz, no. sc-345), STAT2 (Santa Cruz, no. sc-22816), and phosphorylated STAT1 (Y701-specific antibody no. 9171 and S727-specific antibody no. 9177, both from Cell Signaling Technologies). The secondary antibodies used were peroxidase-conjugated goat anti-mouse (no. 115-035-166) or goat anti-rabbit IgG (no. 111-035-144) from Dianova for immunoblotting, and highly cross-adsorbed Alexa Fluor 594- or Alexa Fluor 633-conjugated goat anti-mouse (no. A-11032 or no. A-21052, respectively) and Alexa Fluor 546-conjugated goat anti-rabbit IgG (no. A-11035) from Invitrogen for immunofluorescence.

### ChIP assay

ChIP was performed essentially as described by Nelson *et al.* [217], [218]. Resting cells on a 15-cm dish were cross-linked by treatment with 1% (v/v) formaldehyde for 10 min at 37°C. Isolated chromatin was sonified for 15 min in a Bioruptor (Diagenode; “H” setting, 30 sec on-off interval) and cleared by centrifugation for 20 min at 20,000× g and 4°C. Sheared chromatin from 7×10^6^ cells (0.7 ml) was subjected to immunoprecipitation for 16 h at 4°C with gentle rotation using 10 µg of antibody. Two different polyclonal rabbit antibodies each against STAT1 (no. sc-3454 and sc-346 from Santa Cruz) and STAT2 (no. sc-476 and sc-839 from Santa Cruz) were used. After the antibody incubation step, insoluble material was removed by centrifugation (10 min at 20,000× g and 4°C) and 0.63 ml (90%) supernatant was transferred to a clean pre-chilled tube. Antibody-antigen complexes were isolated by sedimentation following incubation with 60 µl of Protein A Agarose/Salmon Sperm DNA slurry (Millipore) for 60 min at 4°C. PCR-ready DNA was prepared using Chelex-100 and duplicate samples of 5 µl (25% of the final reaction volume) each were used for DNA quantification by qPCR as described above and in recent publications [33], [215]. The PCR primer sequences are listed in Supporting Table S8.

## Supporting Information

Figure S1 Time course qRT-PCR analysis of IFN-β and IFN-γ expression. TetR and TetR-IE1 cells were treated with doxycycline for 3 to 96 h or were left untreated (0 h). Relative mRNA expression levels were determined from 5 µl of undiluted cDNA by qRT-PCR with primers specific for the IFNB and IFNG genes. Results were normalized to TUBB, and means of two biological replicates are shown in comparison to untreated cells (set to 1).
(EPS)

Figure S2 STAT2 knock-down is functionally effective and can down-regulate a *bona fide* STAT2-responsive gene. MRC-5 cells were transfected with control siRNA #149 or STAT2-specific siRNA #152. Four days post transfection cells were treated with IFN-α (10 ng/ml) for 24 h or were left untreated (w/o). Relative mRNA expression levels were determined by qRT-PCR with primers specific for the type I ISGs STAT2 and OAS1. Results were normalized to TUBB and mean values with standard deviations from two biological and two technical replicates are shown. Expression is presented relative to control siRNA-transfected cells without IFN-α stimulation (set to 1).
(EPS)

Table S1 Enrichment of GO “biological process” (GO:0008150) terms (*p*<0.2) in IE1-activated genes.
(DOC)

Table S2 Enrichment of GO “molecular function” (GO:0003674) terms (*p*<0.2) in IE1-activated genes.
(DOC)

Table S3 Enrichment of GO “cellular component” (GO:0005575) terms (*p*<10) in IE1-activated genes.
(DOC)

Table S4 qRT-PCR analysis of IFN responsiveness of IE1-induced genes.
(DOC)

Table S5 Results of GeneChip analysis for IFN genes.
(DOC)

Table S6 qRT-PCR analysis of IFN-β and IFN-γ expression.
(DOC)

Table S7 STAT1 binding sites in the promoter regions of IE1-activated human genes.
(DOC)

Table S8 Oligonucleotides used in this study.
(DOC)

Table S9 siRNAs used in this study.
(DOC)
