# Supplementary material for: Human Cytomegalovirus IE1 Protein Elicits a Type II Interferon-Like Host Cell Response That Depends on Activated STAT1 but Not Interferon-γ
Source: PLoS Pathog. 2011 Apr 14;7(4):e1002016. doi: 10.1371/journal.ppat.1002016 (PMC3077363; doi:10.1371/journal.ppat.1002016)
Supplement: Table S1 — Enrichment of GO “biological process” (GO:0008150) terms (p<0.2) in IE1-activated genes. (DOC) [file ppat.1002016.s003.doc]

**Table S1.** Enrichment of GO “biological process” (GO:0008150) terms (*p* <0.2)^1^ in IE1-activated genes.

| GO term | | *p*-value | Sample frequency^2^ | Swiss-Prot frequency^3^ | Genes |
| --- | --- | --- | --- | --- | --- |
| GO:0002376 | immune system process | 4.80e-10 | 60.0% | 5.8% | CXCL10 IFI44L CCL11 EDN1 GBP2 CXCL9 TAP1 IRF1 HLA-DRA CTSS CD274 TNFSF18 CXCL11 TNFSF4 IDO1 |
| GO:0006955 | immune response | 1.56e-09 | 52.0% | 3.9% | CXCL10 IFI44L CCL11 GBP2 CXCL9 TAP1 HLA-DRA CTSS CD274 TNFSF18 CXCL11 TNFSF4 IDO1 |
| GO:0006954 | inflammatory response | 2.35e-03 | 24.0% | 1.6% | CXCL10 CCL11 CXCL9 CXCL11 TNFSF4 IDO1 |
| GO:0009611 | response to wounding | 2.58e-03 | 28.0% | 2.6% | CXCL10 CCL11 EDN1 CXCL9 CXCL11 TNFSF4 IDO1 |
| GO:0050896 | response to stimulus | 2.59e-03 | 60.0% | 17.7% | CXCL10 IFI44L CCL11 EDN1 GBP2 ANKRD1 CXCL9 TAP1 HLA-DRA CTSS CD274 TNFSF18 CXCL11 TNFSF4 IDO1 |
| GO:0006952 | defense response | 2.94e-03 | 32.0% | 3.9% | CXCL10 CCL11 ANKRD1 CXCL9 TAP1 CXCL11 TNFSF4 IDO1 |
| GO:0042127 | regulation of cell proliferation | 1.83e-02 | 28.0% | 3.5% | CXCL10 HES1 CCL11 EDN1 CD274 TNFSF4 IDO1 |
| GO:0006935 | chemotaxis | 3.90e-02 | 16.0% | 0.8% | CXCL10 CCL11 CXCL9 CXCL11 |
| GO:0042330 | taxis | 3.90e-02 | 16.0% | 0.8% | CXCL10 CCL11 CXCL9 CXCL11 |
| GO:0045321 | leukocyte activation | 5.94e-02 | 20.0% | 1.7% | EDN1 IRF1 CD274 TNFSF4 IDO1 |
| GO:0009605 | response to external stimulus | 6.27e-02 | 28.0% | 4.3% | CXCL10 CCL11 EDN1 CXCL9 CXCL11 TNFSF4 IDO1 |
| GO:0008284 | positive regulation of cell proliferation | 9.04e-02 | 20.0% | 1.9% | CXCL10 HES1 CCL11 EDN1 TNFSF4 |
| GO:0001775 | cell activation | 1.04e-01 | 20.0% | 2.0% | EDN1 IRF1 CD274 TNFSF4 IDO1 |
| GO:0042098 | T cell proliferation | 1.04e-01 | 12.0% | 0.4% | CD274 TNFSF4 IDO1 |
| GO:0042110 | T cell activation | 1.16e-01 | 16.0 | 1.0% | IRF1 CD274 TNFSF4 IDO1 |
| GO:0008283 | cell proliferation | 1.24e-01 | 28.0% | 4.8% | CXCL10 HES1 CCL11 EDN1 CD274 TNFSF4 IDO1 |
| GO:0030335 | positive regulation of cell migration | 1.61e-01 | 12.0% | 0.4% | CXCL10 CCL11 EDN1 |

^1^ Determined using the AmiGO Term Enrichment tool ([http://www.geneontology.org](http://www.geneontology.org/)).

^2^ Frequency among all IE1-activated genes identified in this study.

^3^ Frequency among all proteins present in the Swiss-Prot database (Swiss Institute of Bioinformatics).
