# Supplementary material for: Human Cytomegalovirus IE1 Protein Elicits a Type II Interferon-Like Host Cell Response That Depends on Activated STAT1 but Not Interferon-γ
Source: PLoS Pathog. 2011 Apr 14;7(4):e1002016. doi: 10.1371/journal.ppat.1002016 (PMC3077363; doi:10.1371/journal.ppat.1002016)
Supplement: Table S2 — Enrichment of GO “molecular function” (GO:0003674) terms (p<0.2) in IE1-activated genes. (DOC) [file ppat.1002016.s004.doc]

**Table S2.** Enrichment of GO “molecular function” (GO:0003674) terms (*p* <0.2)^1^ in IE1-activated genes.

| GO term | | *p*-value | Sample frequency^2^ | Swiss-Prot frequency^3^ | Genes |
| --- | --- | --- | --- | --- | --- |
| GO:0005125 | cytokine activity | 6.09e-05 | 24.0% | 0.9% | CXCL10 CCL11 CXCL9 TNFSF18 CXCL11 TNFSF4 |
| GO:0005126 | cytokine receptor binding | 6.33e-05 | 24.0% | 0.9% | CXCL10 CCL11 CXCL9 TNFSF18 CXCL11 TNFSF4 |
| GO:0008009 | chemokine activity | 1.77e-04 | 16.0% | 0.2% | CXCL10 CCL11 CXCL9 CXCL11 |
| GO:0042379 | chemokine receptor binding | 2.87e-04 | 16.0% | 0.2% | CXCL10 CCL11 CXCL9 CXCL11 |
| GO:0001664 | G-protein-coupled receptor binding | 4.23e-04 | 20.0% | 0.6% | CXCL10 CCL11 EDN1 CXCL9 CXCL11 |
| GO:0005102 | receptor binding | 8.71e-03 | 32.0% | 4.5% | CXCL10 CCL11 EDN1 CXCL9 TAP1 TNFSF18 CXCL11 TNFSF4 |
| GO:0003924 | GTPase activity | 1.26e-01 | 16.0% | 1.1% | GBP1 GBP2 GBP4 GBP5 |
| GO:0005164 | tumor necrosis factor receptor binding | 1.49e-01 | 8.0% | 0.1% | TNFSF18 TNFSF4 |

^1^ Determined using the AmiGO Term Enrichment tool ([http://www.geneontology.org](http://www.geneontology.org/)).

^2^ Frequency among all IE1-activated genes identified in this study.

^3^ Frequency among all proteins present in the Swiss-Prot database (Swiss Institute of Bioinformatics).
