# Supplementary material for: Human Cytomegalovirus IE1 Protein Elicits a Type II Interferon-Like Host Cell Response That Depends on Activated STAT1 but Not Interferon-γ
Source: PLoS Pathog. 2011 Apr 14;7(4):e1002016. doi: 10.1371/journal.ppat.1002016 (PMC3077363; doi:10.1371/journal.ppat.1002016)
Supplement: Table S3 — Enrichment of GO “cellular component” (GO:0005575) terms (p<10) in IE1-activated genes. (DOC) [file ppat.1002016.s005.doc]

**Table S3.** Enrichment of GO “cellular component” (GO:0005575) terms (*p* <10)^1^ in IE1-activated genes.

| GO term | | *p*-value | Sample frequency^2^ | Swiss-Prot frequency^3^ | Genes |
| --- | --- | --- | --- | --- | --- |
| GO:0005615 | extracellular space | 1.60e-02 | 28.0% | 3.5% | CXCL10 CCL11 EDN1 CXCL9 TNFSF18 CXCL11 TNFSF4 |
| GO:0044421 | extracellular region part | 1.19e-01 | 28.0% | 4.8% | CXCL10 CCL11 EDN1 CXCL9 TNFSF18 CXCL11 TNFSF4 |
| GO:0042825 | TAP complex | 1.36e+00 | 4.0% | <0.1% | TAP1 |
| GO:0042824 | MHC class I peptide loading complex | 3.41e+00 | 4.0% | <0.1% | TAP1 |
| GO:0005576 | extracellular region | 4.52e+00 | 32.0% | 11.2% | CXCL10 CCL11 EDN1 CXCL9 CTSS TNFSF18 CXCL11 TNFSF4 |

^1^ Determined using the AmiGO Term Enrichment tool ([http://www.geneontology.org](http://www.geneontology.org/)).

^2^ Frequency among all IE1-activated genes identified in this study.

^3^ Frequency among all proteins present in the Swiss-Prot database (Swiss Institute of Bioinformatics).
