# Supplementary material for: Human Cytomegalovirus IE1 Protein Elicits a Type II Interferon-Like Host Cell Response That Depends on Activated STAT1 but Not Interferon-γ
Source: PLoS Pathog. 2011 Apr 14;7(4):e1002016. doi: 10.1371/journal.ppat.1002016 (PMC3077363; doi:10.1371/journal.ppat.1002016)
Supplement: Table S4 — qRT-PCR analysis of IFN responsiveness of IE1-induced genes. (DOC) [file ppat.1002016.s006.doc]

**Table S4.** qRT-PCR analysis of IFN responsiveness of IE1-induced genes.

| Gene symbol | Treatment^1^ | Mean Cq value ± standard deviation^2^ |
| --- | --- | --- |
| TUBB | Mock | 18.50 ± 0.03 |
|  | IFN-α | 18.53 ± 0.04 |
|  | IFN-γ | 18.74 ± 0.05 |
| TNFSF18 | Mock | 25.94 ± 0.24 |
|  | IFN-α | 25.15 ± 0.11 |
|  | IFN-γ | 25.48 ± 0.19 |
| TNFSF4 | Mock | 23.36 ± 0.15 |
|  | IFN-α | 22.96 ± 0.06 |
|  | IFN-γ | 23.99 ± 0.10 |
| SERTAD4 | Mock | 28.74 ± 0.04 |
|  | IFN-α | 28.81 ± 0.09 |
|  | IFN-γ | 28.43 ± 0.08 |
| HES1 | Mock | 29.88 ± 0.15 |
|  | IFN-α | 29.51 ± 0.05 |
|  | IFN-γ | 28.97 ± 0.13 |
| CCDC3 | Mock | 26.94 ± 0.32 |
|  | IFN-α | 26.80 ± 0.17 |
|  | IFN-γ | 27.83 ± 0.17 |
| HBG1 | Mock | >36.00^3^ |
|  | IFN-α | >36.00 |
|  | IFN-γ | >36.00 |
| CCL11 | Mock | 31.08 ± 0.15 |
|  | IFN-α | 31.00 ± 0.18 |
|  | IFN-γ | 30.06 ± 0.11 |

^1^ TetR cells were treated with solvent (Mock), 1000 U/ml IFN-α, or 10 ng/ml IFN-γ.

^2^ Two biological and two technical replicates were analyzed (Cq, quantification cycle).

^3^ Mean Cq value ± standard deviation of TetR-IE1 cells: 26.98 ± 0.13.
