# Supplementary material for: Human Cytomegalovirus IE1 Protein Elicits a Type II Interferon-Like Host Cell Response That Depends on Activated STAT1 but Not Interferon-γ
Source: PLoS Pathog. 2011 Apr 14;7(4):e1002016. doi: 10.1371/journal.ppat.1002016 (PMC3077363; doi:10.1371/journal.ppat.1002016)
Supplement: Table S5 — Results of GeneChip analysis for IFN genes. (DOC) [file ppat.1002016.s007.doc]

**Table S5.** Results of GeneChip analysis for IFN genes.

| Gene | | Maximum fold increase | | | | |
| --- | --- | --- | --- | --- | --- | --- |
|  | | w/o induction | 24 h post induction | | 72 h post induction | |
| ID | Symbol^1^ | IE1-/TetR- | IE1+/TetR+ | IE1+/IE1- | IE1+/TetR+ | IE1+/IE1- |
| 3439 | IFNA1 | 1.07 | -1.02 | 1.21 | -1.29 | -1.16 |
| 3440 | IFNA2 | 1.10 | -1.09 | -1.13 | 1.06 | -1.04 |
| 3441 | IFNA4 | -1.06 | -1.03 | -1.08 | 1.01 | -1.01 |
| 3442 | IFNA5 | 1.04 | 1.09 | 1.17 | -1.01 | 1.08 |
| 3443 | IFNA6 | 1.00 | 1.13 | 1.31 | 1.12 | -1.03 |
| 3445 | IFNA8 | 1.03 | -1.04 | -1.01 | -1.03 | 1.00 |
| 3446 | IFNA10 | -1.04 | 1.04 | 1.06 | -1.05 | -1.02 |
| 3447 | IFNA13 | 1.03 | 1.07 | 1.04 | -1.08 | -1.05 |
| 3448 | IFNA14 | -1.07 | -1.05 | 1.06 | -1.03 | -1.17 |
| 3449 | IFNA16 | 1.15 | -1.22 | -1.16 | -1.09 | 1.03 |
| 3451 | IFNA17 | -1.03 | -1.05 | -1.02 | 1.01 | 1.04 |
| 3452 | IFNA21 | 1.03 | -1.05 | -1.15 | -1.10 | -1.07 |
| 3456 | IFNB1 | 1.06 | -1.05 | 1.00 | 1.14 | 1.03 |
| 338376 | IFNE | -1.15 | -1.11 | -1.32 | 1.01 | 1.13 |
| 56832 | IFNK | 1.00 | 1.00 | -1.13 | -1.07 | -1.10 |
| 3467 | IFNW1 | 1.08 | -1.07 | 1.14 | -1.25 | -1.13 |
| 3458 | IFNG | -1.04 | -1.04 | -1.12 | 1.01 | 1.03 |
| 282616 | IL28A | 1.09 | -1.01 | 1.13 | -1.01 | -1.09 |
| 282617 | IL28B | -1.11 | 1.12 | 1.12 | 1.11 | 1.28 |
| 282618 | IL29 | 1.01 | -1.15 | -1.06 | -1.06 | -1.09 |

^1^ The IFNA7 gene is not represented on the Affymetrix GeneChip Human Gene 1.0 ST Arrays used in this study.

IE1-, non-induced TetR-IE1 cells; TetR-, non-induced TetR cells; IE1+, doxycycline-treated TetR-IE1 cells; TetR+, doxycycline-treated TetR cells.
