# Supplementary material for: Human Cytomegalovirus IE1 Protein Elicits a Type II Interferon-Like Host Cell Response That Depends on Activated STAT1 but Not Interferon-γ
Source: PLoS Pathog. 2011 Apr 14;7(4):e1002016. doi: 10.1371/journal.ppat.1002016 (PMC3077363; doi:10.1371/journal.ppat.1002016)
Supplement: Table S6 — qRT-PCR analysis of IFN-β and IFN-γ expression. (DOC) [file ppat.1002016.s008.doc]

**Table S6.** qRT-PCR analysis of IFN-β and IFN-γ expression.

| Sample^1,2,3,4^ | | | Cq value^5^ | | | | | | | | |
| --- | --- | --- | --- | --- | --- | --- | --- | --- | --- | --- | --- |
|  |  |  | IFNB | | | IFNG | | | TUBB | | |
| +RT | TetR | w/o | 31.89 | 31.33 | 31.56 | 33.74 | *33.47* | *33.92* | 15.62 | 15.64 | 15.96 |
|  |  | Doxy, 24 h | 31.70 | 32.26 | 31.88 | 33.61 | 33.60 | *33.76* | 15.77 | 15.86 | 15.95 |
|  |  | Doxy, 72 h | 31.67 | 31.43 | 31.96 | *33.83* | *34.15* | *34.00* | 15.86 | 15.70 | 15.89 |
|  | TetR-IE1 | w/o | 31.12 | 31.31 | 32.17 | *33.60* | *33.80* | 34.49 | 15.65 | 15.53 | 15.82 |
|  |  | Doxy, 24 h | 31.87 | 32.02 | 31.89 | 34.59 | 33.76 | 34.52 | 15.42 | 15.71 | 15.57 |
|  |  | Doxy, 72 h | 31.05 | 30.92 | 31.50 | 34.34 | *33.94* | 34.99 | 15.18 | 15.31 | 15.67 |
| -RT | TetR | w/o | 35.79 | 37.58 | 37.59 | *>46.00* | *>46.00* | *35.57* | 35.90 | 37.07 | 35.94 |
|  |  | Doxy, 24 h | *>46.00* | *>46.00* | 36.06 | *>46.00* | 36.56 | *37.59* | 35.07 | 33.98 | *30.69* |
|  |  | Doxy, 72 h | *>46.00* | *>46.00* | 36.63 | *>46.00* | *37.47* | 36.31 | 35.86 | 37.06 | *29.30* |
|  | TetR-IE1 | w/o | 35.65 | 36.59 | 37.69 | *>46.00* | 36.68 | *>46.00* | 35.60 | 35.09 | 36.14 |
|  |  | Doxy, 24 h | *>46.00* | 37.66 | *>46.00* | 36.60 | 36.80 | *>46.00* | 34.70 | 34.82 | 34.97 |
|  |  | Doxy, 72 h | 37.53 | 36.25 | *>46.00* | *>46.00* | *>46.00* | *>46.00* | 34.66 | 35.87 | *29.94* |
| +RT | MRC-5 | hCMV | 26.47 | 26.28 | − | − | − | − | 15.72 | 15.92 | − |
|  | PBMC | PMA/IM | − | − | − | 14.94 | 14.78 | − | 23.47 | 23.17 | − |

^1^ Undiluted cDNA (5 µl) was used for qRT-PCR.

^2^ TetR and TetR-IE1 cells were treated with doxycycline (Doxy) for 24 h or 72 h or were left untreated (w/o).

^3^ MRC-5 cells were infected with TNwt at 1 PFU/cell for 6 h.

^4^ PBMC were stimulated with 1 µg/ml phorbol 12-myristate 13-acetate (PMA) and 1 µg/ml ionomycin (IM) for 6 h.

^5^ Cq values from up to three biological replicates are shown; samples for which melting curve analysis revealed formation of non-specific products are italicized.

+RT, with reverse transcriptase; -RT, without reverse transcriptase; PBMC, peripheral blood mononuclear cells; −, not determined.
