# Supplementary material for: Human Cytomegalovirus IE1 Protein Elicits a Type II Interferon-Like Host Cell Response That Depends on Activated STAT1 but Not Interferon-γ
Source: PLoS Pathog. 2011 Apr 14;7(4):e1002016. doi: 10.1371/journal.ppat.1002016 (PMC3077363; doi:10.1371/journal.ppat.1002016)
Supplement: Table S7 — STAT1 binding sites in the promoter regions of IE1-activated human genes. (DOC) [file ppat.1002016.s009.doc]

**Table S7.** STAT1 binding sites in the promoter regions of IE1-activated human genes.

| Gene symbol | Gene location | | | STAT1 ChIP-seq peaks^2^ | | |
| --- | --- | --- | --- | --- | --- | --- |
|  | Chromosome | Strand | Transcription start site(s)^1^ | Start | End | Height |
| IFI44L | 1 | + | 78,858,676 | **78,857,794** | **78,858,890** | **12** |
| GBP1 | 1 | - | 89,303,631 | **89,303,023** | **89,304,211** | **63** |
| GBP2 | 1 | - | 89,364,387 | **89,363,912** | **89,364,866** | **38** |
| GBP4 | 1 | - | 89,437,150 | – | – | – |
| GBP5 | 1 | - | 89,511,132 | **89,512,393** | **89,514,664** | 972 |
| CTSS | 1 | - | 149,004,929 | **149,003,610** | **149,005,566** | **284** |
| TNFSF18 | 1 | - | 171,286,726 | – | – | – |
| TNFSF4 | 1 | - | 171,443,094 | – | – | – |
| SERTAD4 | 1 | + | 208,472,818 | – | – | – |
| HES1 | 3 | + | 195,336,628 | **195,334,937** | **195,338,850** | **18** |
| CXCL9 | 4 | - | 77,147,665 | **77,147,440** | **77,148,273** | **16** |
| CXCL10 | 4 | - | 77,163,674 | – | – | – |
| CXCL11 | 4 | - | 77,176,257 | – | – | – |
| IRF1 | 5 | - | 131,854,364 | **131,849,355** | **131,857,028** | **1399** |
|  |  |  |  | 131,853,554 | 131,854,809 | 21 |
| EDN1 | 6 | + | 12,398,515 | 12,397,038 | 12,398,942 | 11 |
| HLA-DRA | 6 | + | 32,515,625 | – | – | – |
| TAP1 | 6 | - | 32,929,726 | **32,927,703** | **32,930,873** | **729** |
|  |  |  |  | 32,928,001 | 32,930,633 | 13 |
| IDO1 | 8 | + | 39,890,485 | **39,888,986** | **39,890,831** | **56** |
| CD274 | 9 | + | 5,440,559 | – | – | – |
| CCDC3 | 10 | - | 13,083,710 | – | – | – |
| IFIT2 | 10 | + | 91,051,686 | **91,051,480** | **91,052,251** | **17** |
| IFIT3 | 10 | + | 91,077,733 | **91,077,219** | **91,078,543** | **21** |
|  |  |  |  | 91,077,222 | 91,078,265 | 12 |
|  |  |  | 91,082,283 | – | – | – |
| ANKRD1 | 10 | - | 92,671,012 | **92,670,630** | **92,671,623** | **38** |
| HBG1 | 11 | - | 5,227,663 | – | – | – |
| CCL11 | 17 | + | 29,636,800 | – | – | – |

^1^ Transcription start sites were extracted from the March 2006 human NCBI36 (hg18) assembly.

^2^ ChIP-sequencing (ChIP-seq) data were retrieved from Robertson G, Hirst M, Bainbridge M, Bilenky M, Zhao Y, Zeng T, Euskirchen G, Bernier B, Varhol R, Delaney A, Thiessen N, Griffith OL, He A, Marra M, Snyder M, Robertson JS (2007) [Genome-wide profiles of STAT1 DNA association using chromatin immunoprecipitation and massively parallel sequencing.](http://www.ncbi.nlm.nih.gov/pubmed/17558387) Nat Methods 4: 651-657. ChIP-seq peaks found in unstimulated cells are shown in regular font style, peaks found after IFN-γ stimulation are shown in bold.
