# Supplementary material for: Human Cytomegalovirus IE1 Protein Elicits a Type II Interferon-Like Host Cell Response That Depends on Activated STAT1 but Not Interferon-γ
Source: PLoS Pathog. 2011 Apr 14;7(4):e1002016. doi: 10.1371/journal.ppat.1002016 (PMC3077363; doi:10.1371/journal.ppat.1002016)
Supplement: Table S8 — Oligonucleotides used in this study. (DOC) [file ppat.1002016.s010.doc]

**Table S8.** Oligonucleotides used in this study.

| Oligonucleotide # | Sequence (5‘→3‘) | Use |
| --- | --- | --- |
| 103 | GGAGGAAAAAGAGTCCTCTA | IFIT2 ChIP analysis |
| 104 | AGCTGCACTCTTCAGAAA | IFIT2 ChIP analysis |
| 109 | TCTTCATGCTCCAGACGTAC | MxA mRNA quantification |
| 110 | CCAGCTGTAGGTGTCCTTG | MxA mRNA quantification |
| 117 | ATTCCCTGTGCACCGTAG | IRF1 mRNA quantification |
| 118 | CCATATCCACCATGATGC | IRF1 mRNA quantification |
| 121 | ACGGTATGCTTGGAACGATTG | IFIT2 mRNA quantification |
| 122 | AACCCAGAGTGTGGCTGATG | IFIT2 mRNA quantification |
| 136 | GCGGCAGATCTCATGCGGCTCACCTCGTCAATCTTG | pGS284-TNIE1*kanlac*Z cloning |
| 137 | GCGGCGAATTCGCGGCCGCTTAATTAAACTATTGTATATATATATCAGTTACTG | pGS284-TNIE1*kanlac*Z cloning |
| 139 | GCGGCGAATTCGCGGCCGCTTAATTAACAACACATATAAGTATCCGTC | pGS284-TNIE1*kanlac*Z cloning |
| 140 | GCGGCGCATGCGATCTCCACGCGAATCTCGGGTAC | pGS284-TNIE1*kanlac*Z cloning |
| 155 | gagtccgaagccgaactgcag | pGS248-TNMIE cloning |
| 156 | gcggcgcggccgcgatctccacgcgaatctcg | pGS248-TNMIE cloning |
| 292 | CCTAGTGTGGATGACCTA | IE1 mRNA quantification |
| 293 | GTGACACCAGAGAATCAG | IE1 mRNA quantification |
| 294 | CACCAAAGACACGTCGTT | hCMV genome quantification |
| 295 | GTCCTTTGCGACCAGAAT | hCMV genome quantification |
| 348 | TTGCAAGATCTCTCTGTCCTTCAGAGACTGACA | pGS284-TNMIE*dl*IE1 cloning |
| 349 | GAGTATAATATAGAGTATACATCACATACATGTCAACAGA | pGS284-TNMIE*dl*IE1 cloning |
| 350 | TCTGTTGACATGTATGTGATGTATACTCTATATTATACTC | pGS284-TNMIE*dl*IE1 cloning |
| 351 | TTGCAGCGGCCGCCATCTAACACCTGGTGCATACTG | pGS284-TNMIE*dl*IE1 cloning |
| 363 | TATCAGCAGTACCAGGATGC | TUBB ChIP analysis |
|  |  | TUBB mRNA quantification |
| 364 | TGAGAAGCCTGAGGTGATG | TUBB ChIP analysis |
|  |  | TUBB mRNA quantification |
| 457 | CTGGACAATCTCACACCTTAC | STAT2 mRNA quantification |
| 458 | GAACATCTTGGAGCAATGTC | STAT2 mRNA quantification |
| 483 | TTGCAAAGCTTATGGAGTCCTCTGCCAAGAGAAAG | IE1 cloning |
| 484 | TTGCAGAATTCTTACTGGTCAGCCTTGCTTCTAG | IE1 cloning |
| 492 | TCGACAGTCAGCCGCATCT | GAPDH ChIP analysis |
| 493 | CTAGCCTCCCGGGTTTCTCT | GAPDH ChIP analysis |
| 494 | CAAGGCAAAGCGAAATTGGT | RPL30 ChIP analysis |
| 495 | GCCCGTTCAGTCTCTTCGATT | RPL30 ChIP analysis |
| 531 | GAGTGCAAGGAACCCCAGTAGT | CXCL9 mRNA quantification |
| 532 | GGTGGATAGTCCCTTGGTTGGT | CXCL9 mRNA quantification |
| 533 | TCCACGTGTTGAGATCATTGC | CXCL10 mRNA quantification |
| 534 | TCTTGATGGCCTTCGATTCTG | CXCL10 mRNA quantification |
| 535 | CAAGGCTTCCCCATGTTCA | CXCL11 mRNA quantification |
| 536 | CCCAGGGCGTATGCAAAGA | CXCL11 mRNA quantification |
| 537 | GCTCCAAGCAGTCCTTTCAC | GBP4 mRNA quantification |
| 538 | GTGGTGGCTCATGCCTAAAT | GBP4 mRNA quantification |
| 547 | GGTGGAAGAGGGACAAACAA | GBP4 ChIP analysis |
| 548 | CCAGCCGGATTTACAGACAT | GBP4 ChIP analysis |
| 596 | TTCAGGAAGACCCAATCCAG | STAT1 mRNA quantification |
| 597 | TGAATATTCCCCGACTGAGC | STAT1 mRNA quantification |
| 612 | gcaggccacttttggaagta | TAP1 ChIP analysis |
| 613 | ggaaagtcccaggaacagg | TAP1 ChIP analysis |
| 614 | TTGGCTGGAGGTTAAAATGC | OAS1 ChIP analysis |
| 615 | AGCTTGGACTGCTGTTGGTT | OAS1 ChIP analysis |
| 616 | GCAGCTCATGGAGAAAAAGG | TAP1 mRNA quantification |
| 617 | AAGGCTTTCATTCTGGAGCA | TAP1 mRNA quantification |
| 618 | TGTGCCAAAGGCTATCAGTG | CXCL9 ChIP analysis |
| 619 | CTGGGGGAAACCCTAGTCTC | CXCL9 ChIP analysis |
| 644 | GATGGTGGCCTCTCTGACTT | TNFSF4 mRNA quantification |
| 645 | ATCAGTTCTCCGCCATTCAC | TNFSF4 mRNA quantification |
| 646 | GTGTTCAGGAAAAGGCCAAA | SERTAD4 mRNA quantification |
| 647 | GTCATTTCCCACAGGTTCGT | SERTAD4 mRNA quantification |
| 648 | TTGCTTTCCTCATTCCCAAC | HES1 mRNA quantification |
| 649 | GTGTAGACGGGGATGACAGG | HES1 mRNA quantification |
| 650 | CAGAAGGCCTTGTTTGAGGA | CCDC3 mRNA quantification |
| 651 | GCCACTTTCTGCTGCAGTTT | CCDC3 mRNA quantification |
| 652 | CCTGGCAGAAGATGGTGACT | HBG1 mRNA quantification |
| 653 | AGCTCTGAATCATGGGCAGT | HBG1 mRNA quantification |
| 654 | GACCAAACTGGCCAAGGATA | CCL11 mRNA quantification |
| 655 | TCCTGCACCCACTTCTTCTT | CCL11 mRNA quantification |
| 656 | GCAAATCCCCAATTCATCTC | TNFSF18 mRNA quantification |
| 657 | ATGTGCTGAAGGGAATGAGG | TNFSF18 mRNA quantification |
| 660 | GAACTGGTTCACTATAGTCGCAGAGAGTCTGCAGCAAGTT | STAT1* mutagenesis |
| 661 | AACTTGCTGCAGACTCTCTGCGACTATAGTGAACCAGTTC | STAT1* mutagenesis |
| 662 | GGCCCTAAAGGAACTGGATTTATCAAGACTGAGTTGATT | STAT1*Y701F mutagenesis |
| 663 | AATCAACTCAGTCTTGATAAATCCAGTTCCTTTAGGGCC | STAT1*Y701F mutagenesis |
| 664 | AACCTGCTCCCCATGGCTCCTGAGGAGTTTG | STAT1*S727A mutagenesis |
| 665 | CAAACTCCTCAGGAGCCATGGGGAGCAGGTT | STAT1*S727A mutagenesis |
| 684 | ccctcagtccgtgagtttgt | IDO1 mRNA quantification |
| 685 | gctttcacacaggcgtcata | IDO1 mRNA quantification |
| 688 | CTGGCGGCTATAAACCTAACC | OAS1 mRNA quantification |
| 689 | GTTCTGTGAAGCAGGTGGAGA | OAS1 mRNA quantification |
| 690 | ATGATGGAAAGCGAACAAGG | PKR mRNA quantification |
| 691 | CAGCAAGAATTAGCCCCAAA | PKR mRNA quantification |
