# Supplementary material for: Human Cytomegalovirus IE1 Protein Elicits a Type II Interferon-Like Host Cell Response That Depends on Activated STAT1 but Not Interferon-γ
Source: PLoS Pathog. 2011 Apr 14;7(4):e1002016. doi: 10.1371/journal.ppat.1002016 (PMC3077363; doi:10.1371/journal.ppat.1002016)
Supplement: Table S9 — siRNAs used in this study. (DOC) [file ppat.1002016.s011.doc]

**Table S9.** siRNAs used in this study.

| siRNA # | Sequence^1^ | Company (catalog no.) | Use |
| --- | --- | --- | --- |
| 6 | AAGAUUCUGCAGCAUUUCCCACUCC | Invitrogen (45-1826) | STAT2 knock-down |
| 140 | UUCGUGUAGGGUUCAACCGdCdA | Ambion (s278) | STAT1 knock-down |
| 146 | UCCGCAACUAUAGUGAACCdAdG | Ambion (s279) | STAT1 knock-down |
| 149 | – | Ambion (4390843) | Negative control |
| 152 | UUAGAGACCACAAUGAGCCdTdG | Ambion (s13528) | STAT2 knock-down |

^1^ guide (antisense) strand (5‘→3‘).

d, desoxy.
